# Engineered reduction of *S*-adenosylmethionine alters lignin in sorghum

**DOI:** 10.1186/s13068-024-02572-8

**Published:** 2024-10-15

**Authors:** Yang Tian, Yu Gao, Halbay Turumtay, Emine Akyuz Turumtay, Yen Ning Chai, Hemant Choudhary, Joon-Hyun Park, Chuan-Yin Wu, Christopher M. De Ben, Jutta Dalton, Katherine B. Louie, Thomas Harwood, Dylan Chin, Khanh M. Vuu, Benjamin P. Bowen, Patrick M. Shih, Edward E. K. Baidoo, Trent R. Northen, Blake A. Simmons, Robert Hutmacher, Jackie Atim, Daniel H. Putnam, Corinne D. Scown, Jenny C. Mortimer, Henrik V. Scheller, Aymerick Eudes

**Affiliations:** 1 Joint BioEnergy Institute, Emeryville, CA 94608 USA; 2 Environmental Genomics and Systems Biology Division, Lawrence Berkeley National Laboratory, One Cyclotron Road, MS 978R4468, Berkeley, CA 94720 USA; 3 Department of Energy System Engineering, Karadeniz Technical University, 61830 Trabzon, Turkey; 4 Department of Chemistry, Recep Tayyip Erdogan University, 53100 Rize, Turkey; 5 Department of Bioresource and Environmental Security, Sandia National Laboratories, Livermore, CA 94550 USA; 6 Forage Genetics International, West Salem, WI 54669 USA; 7 Department of Plant Sciences, University of California-Davis, Davis, CA 95616 USA; 8 Joint Genome Institute, Lawrence Berkeley National Laboratory, Berkeley, CA 94720 USA; 9 Rausser College of Natural Resources, University of California-Berkeley, Berkeley, CA 94720 USA; 10 Department of Plant and Microbial Biology, University of California-Berkeley, Berkeley, CA 94720 USA; 11 Biological Systems and Engineering Division, Lawrence Berkeley National Laboratory, Berkeley, CA 94720 USA; 12 University of California, Agriculture and Natural Resources, Kearney Agricultural Research and Extension Center, Parlier, CA 93648 USA; 13 Energy & Biosciences Institute, University of California-Berkeley, Berkeley, CA 94720 USA; 14 Energy Analysis and Environmental Impacts Division, Lawrence Berkeley National Laboratory, Berkeley, CA 94720 USA; 15 School of Agriculture, Food, and Wine, University of Adelaide, Glen Osmond, South Australia Australia

**Keywords:** Cell wall, Monolignols, Saccharification, *O*-methyltransferases, Bioenergy crop

## Abstract

**Background:**

Lignin is an aromatic polymer deposited in secondary cell walls of higher plants to provide strength, rigidity, and hydrophobicity to vascular tissues. Due to its interconnections with cell wall polysaccharides, lignin plays important roles during plant growth and defense, but also has a negative impact on industrial processes aimed at obtaining monosaccharides from plant biomass. Engineering lignin offers a solution to this issue. For example, previous work showed that heterologous expression of a coliphage *S*-adenosylmethionine hydrolase (AdoMetase) was an effective approach to reduce lignin in the model plant Arabidopsis. The efficacy of this engineering strategy remains to be evaluated in bioenergy crops.

**Results:**

We studied the impact of expressing AdoMetase on lignin synthesis in sorghum (*Sorghum bicolor* L. Moench). Lignin content, monomer composition, and size, as well as biomass saccharification efficiency were determined in transgenic sorghum lines. The transcriptome and metabolome were analyzed in stems at three developmental stages. Plant growth and biomass composition was further evaluated under field conditions. Results evidenced that lignin was reduced by 18% in the best transgenic line, presumably due to reduced activity of the *S*-adenosylmethionine-dependent *O*-methyltransferases involved in lignin synthesis. The modified sorghum features altered lignin monomer composition and increased lignin molecular weights. The degree of methylation of glucuronic acid on xylan was reduced. These changes enabled a ~20% increase in glucose yield after biomass pretreatment and saccharification compared to wild type. RNA-seq and untargeted metabolomic analyses evidenced some pleiotropic effects associated with *AdoMetase* expression. The transgenic sorghum showed developmental delay and reduced biomass yields at harvest, especially under field growing conditions.

**Conclusions:**

The expression of *AdoMetase* represents an effective lignin engineering approach in sorghum. However, considering that this strategy potentially impacts multiple *S*-adenosylmethionine-dependent methyltransferases, adequate promoters for fine-tuning *AdoMetase* expression will be needed to mitigate yield penalty.

**Supplementary Information:**

The online version contains supplementary material available at 10.1186/s13068-024-02572-8.

## Background

**Fig. 1:**
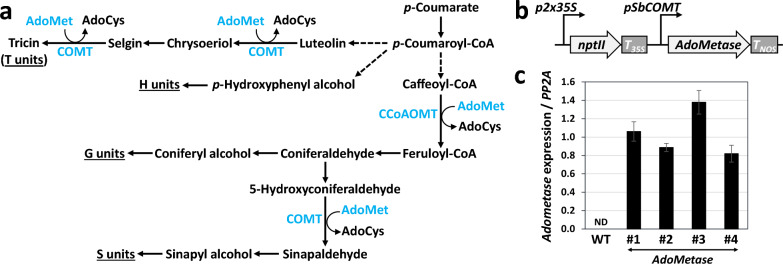
Expression of *AdoMetase* in sorghum. **a** Simplified lignin biosynthetic pathway showing the involvement of the two AdoMet-utilizing enzymes COMT and CCoAOMT. Dashed arrows denote multiple steps. AdoCys, *S*-adenosylhomocysteine. **b** Construct used for sorghum transformation. Genetic elements in the T-DNA include a selectable marker gene for kanamycin resistance (*nptII*) placed between a duplicated 35S promoter (*p2* × *35S*) and a terminator (*T*_*35S*_) from the cauliflower mosaic virus, as well as the *AdoMetase* synthetic gene located between the sorghum COMT gene promoter (*pSbCOMT*) and the *Agrobacterium tumefaciens* nopaline synthase gene terminator (*T*_*NOS*_). **c** *AdoMetase* expression in four independent transgenic lines. The level of *AdoMetase* expression compared to the *PP2A* gene is shown. First-strand cDNA obtained from wild type (WT) were used as negative control. Values are means ± SD of four biological replicates (*n* = 4 plants). *ND* not detected

Dedicated bioenergy crops and residues from agriculture and forestry represent a renewable source of fermentable sugars for the production of advanced bioproducts and biofuels [1, 2]. A large fraction of the sugars contained in lignocellulosic biomass derive from the cell wall polymers cellulose and hemicellulose, which are surrounded by a protective, hydrophobic and resistant polymer called lignin [3]. The main units in lignin arise from the oxidative polymerization of methoxylated hydroxycinnamyl alcohols synthesized from *p*-coumarate [4]. These include coniferyl and sinapyl alcohols that generate the guaiacyl (G) and syringyl (S) lignin units, respectively. The presence of small amounts of *p*-hydroxyphenyl alcohol (H units) in lignin has also been documented, in addition to the occurrence of the flavone tricin (T units) in certain plant species (Fig. 1a), as well as a range of other phenolic compounds that form rare and atypical lignin monomers [5–7]. Several approaches have been proposed to make the conversion of lignocellulosic feedstocks into bioproducts cheaper and more efficient [8]. For example, the manipulation of lignin content and monomer composition via genetic engineering represents an important milestone to reduce lignin recalcitrance and facilitate the enzymatic deconstruction of cellulose and hemicellulose into simple sugars [9, 10].

*S*-adenosylmethionine (AdoMet) is a universal methyl-group donor produced in the cytosol and needed for the methylation of a large number of metabolites as well as a precursor for the synthesis of polyamines, nicotianamine, 5ʹ-deoxyadenosyl radicals, and ethylene [11, 12]. The lignin biosynthetic pathway contains two enzymes that utilize AdoMet for transmethylation reactions: caffeoyl-CoA *O*-methyltransferase (CCoAOMT) is involved in the 3-*O*-methylation step that produces feruloyl-CoA, and caffeic acid *O*-methyltransferase (COMT) methylates 5-hydroxyconiferaldehyde to produce sinapaldehyde. COMT is a promiscuous enzyme that has the ability to catalyze both 5-*O*- and 3-*O*-methylation of several lignin pathway intermediates including acids and alcohols, suggesting a functional redundancy between CCoAOMT and COMT in lignin synthesis [13]. In addition, a few studies have demonstrated the role of COMT in the synthesis of tricin [14–16] (Fig. 1a). As an important reaction in one-carbon metabolism, AdoMet is regenerated from ATP and methionine (Met) by AdoMet synthetase via the Met cycle. The characterization of several mutants in Arabidopsis, sorghum, and maize highlighted the importance of AdoMet as a methyl donor for lignin biosynthesis. For example, mutation in one of the AdoMet synthetase genes in Arabidopsis results in concomitant reductions of AdoMet and lignin [17]. Moreover, the alteration of genes responsible for the synthesis of 5-methyltetrahydrofolate—a cofactor used by Met synthase for the synthesis of Met and AdoMet regeneration—reduces the lignin content in maize, sorghum, switchgrass, and Arabidopsis [18–22]. In particular, these genetic modifications affect folylpolyglutamate synthase (FPGS) and 5-methyltetrahydrofolate reductase (MTHFR) in the folate cycle that mediates one-carbon metabolism. For example, both the *brown midrib4* (*bm4*) gene in maize and the *brown midrib19* (*bmr19*) gene in sorghum encode an FPGS. The corresponding *bm4* and *bmr19* mutants show an increased lignin S/G ratio, as also observed in the Arabidopsis *fpgs* mutant [18, 19, 21, 23]. Silencing of *FPGS* in switchgrass reduced the lignin content and resulted in a significant biomass yield penalty when grown in the field [20]. A mutation in the *brown midrib2* (*bm2*) gene that encodes a MTHFR in maize also results in a higher lignin S/G ratio [22, 23].

We previously demonstrated in Arabidopsis that the heterologous expression of AdoMet hydrolase from *E. coli* phage (AdoMetase, EC 3.13.2.2) was an effective approach to reduce AdoMet and lignin [24]. This engineering strategy is potentially transferable to any crop amenable to genetic transformation and could enable the modification of lignin without any prior knowledge of the CCoAOMT/COMT genes involved in lignification or the genes that participate in AdoMet synthesis. By using tissue-specific promoters, this approach also offers an opportunity for reducing AdoMet preferentially in lignifying tissues while avoiding pleiotropic effects resulting from the modification of other metabolic pathways that consume AdoMet. The major demand for one-carbon units is allocated for lignin and polysaccharide biosynthesis, and reducing AdoMet specifically in the tissues that produce lignified secondary cell walls may have limited impact on cellular functions that have lower AdoMet demand [21, 25].

In the current study, we assessed the impact of expressing *AdoMetase* in the bioenergy crop sorghum (*Sorghum bicolor* L. Moench) using the promoter of a *COMT* gene (*bmr12*) involved in lignification. The transgenic lines have reductions in lignin content, show altered lignin monomer composition, and feature increased lignin molecular weights. These modifications result in improvements of biomass saccharification efficiency compared to the wild type, under both greenhouse and field growth conditions. The analysis of the transcriptome and metabolome in stems of the transgenic lines revealed some changes in metabolic processes other than lignin biosynthesis, indicating some pleiotropic effects associated with *AdoMetase* expression, which may explain the reduction in biomass yield and developmental delay observed in some lines.

## Results

### Generation of sorghum expressing *AdoMetase*

**Fig. 2:**
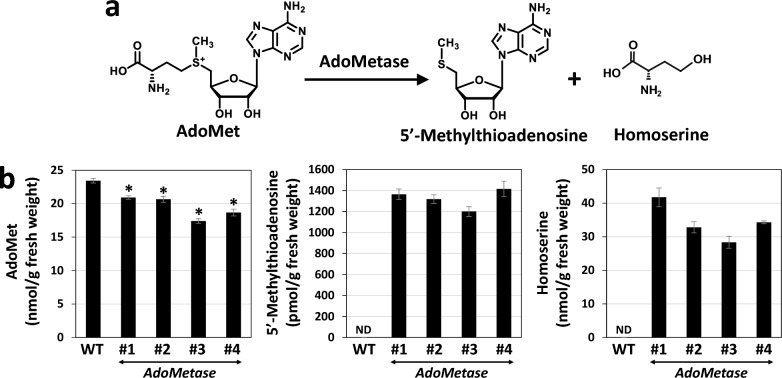
Adometase enzymatic reaction (**a**) and quantification of AdoMet, 5ʹ-methylthioadenosine, and homoserine in 3-week-old seedlings from wild-type (WT) and *AdoMetase* lines (**b**). Values are means ± SD of five biological replicates (*n* = 5 plants). Asterisks indicate a significant difference from the WT using the unpaired Student’s *t* test (*P < 0.05). *ND* not detected

A construct containing the promoter region of the sorghum *COMT* gene (*bmr12*) followed by a codon-optimized *AdoMetase* open reading frame was built for *Agrobacterium*-mediated sorghum transformation (Fig. 1b). The *pSbCOMT* promoter was chosen in an attempt to express *AdoMetase* preferentially in lignifying tissues. Several primary T0 transformants were regenerated from calli and analyzed by Taqman real-time PCR to identify lines containing a single copy of the transgene. Four of these single-copy events were grown in the T1 generation and wild-type (WT) segregants and homozygous plants were identified. T2 seeds from one WT segregant plant from each line were pooled and used as controls for further experiments. The expression of *AdoMetase* in each transgenic line was confirmed by qPCR using first-strand cDNA synthesized from total RNAs extracted from stems of 3-week-old plants (Fig. 1c). Consistent with AdoMetase activity, the analysis of metabolites extracted from 3-week-old seedlings showed in transgenics some reductions of AdoMet (by 11–26%) and the presence of the two AdoMetase products 5ʹ-methylthioadenosine and homoserine (Fig. 2). Measurements of growth parameters including the number of days to panicle emergence, number of flowering tillers, height of the main tiller, and dry weight from stover at plant maturity revealed that transgenics were shorter and had delayed flowering compared to the WT. In addition, lines *AdoMetase* #3 and #4 had reduced biomass yield (Table 1).

**Table 1:** Growth parameters of wild-type and *AdoMetase* lines

	Panicle emergence (days)	Number of flowering tillers (*n*)	Main tiller height (cm)	Stover dry weight (g)
Wild type	67.5 (1.4)	5.0 (0.7)	81.3 (2.8)	170.6 (4.4)
*AdoMetase* #1	81.0 (2.1)*	4.3 (0.5)	53.8 (1.8)*	154.9 (7.9)
*AdoMetase* #2	78.5 (1.2)*	5.8 (0.6)	74.2 (4.5)*	174.0 (16.2)
*AdoMetase* #3	76.7 (0.4)*	5.8 (0.7)	68.4 (3.7)*	129.7 (6.4)*
*AdoMetase* #4	76.2 (1.3)*	5.3 (0.7)	69.7 (2.2)*	145.8 (6.6)*

Values in brackets are the SE from six biological replicates (*n* = 6 plants)

Asterisks (bold values) indicate a significant difference from the wild type using the unpaired Student’s *t* test (*P < 0.05)

### Cell wall composition analysis

The chemical composition of extractive-free cell wall residues obtained from leaf and stem biomass was determined using a two-step acid hydrolysis procedure. The content of acid-insoluble lignin was significantly reduced in the *AdoMetase* lines #1 (−18%), #2 (−11%), and #4 (−7%). Similar amount of glucose and xylose was obtained from the cell wall of the different lines, indicating that cellulose and xylan contents do not differ between WT and transgenics (Table 2). However, glucuronic acid was significantly increased in the *AdoMetase* lines #1 and #2, whereas 4-*O*-methylglucuronic acid was decreased in the *AdoMetase* lines #2 and #4. Glucuronic acid typically occurs as side chains on the xylan backbone and its degree of methylation (or 4-*O*-methylglucuronic/glucuronic acid ratio) was significantly reduced in the *AdoMetase* lines #1, #2, and #4 compared to WT (Table 2). Arabinose, which is also found as side chains on xylan, was not consistently changed among the four *AdoMetase* lines compared to WT (Table 2). The aromatics *p*-coumarate and ferulate were released from cell wall residues by mild alkaline hydrolysis and quantified. All transgenic lines showed reductions of ferulate (−8 to 15%) and the *AdoMetase* line #1 had 15% less *p*-coumarate, although the biosynthesis of the latter does not require AdoMet (Table 2).

**Table 2:** Chemical composition of cell walls isolated from wild-type and *AdoMetase* lines grown in the greenhouse until full maturity

	*AdoMetase* lines				
	Wild type	#1	#2	#3	#4
Glucose	326.7 (1.7)	344.5 (7.1)	340.8 (5.9)	329.7 (2.6)	334.1 (2.9)
Xylose	218.5 (2.2)	229.7 (4.6)	218.3 (4.6)	217.7 (0.2)	220.4 (2.3)
Arabinose	34.5 (0.2)	39.8 (1.4)*	35.1 (0.8)	33.0 (0.5)*	35.1 (0.2)
Acid-insoluble lignin	138.3 (2.6)	113.6 (3.9)*	123.4 (3.1)*	132.6 (4.1)	129.2 (1.6)*
Acid-soluble lignin	16.3 (0.1)	17.9 (0.2)*	17.8 (0.2)*	16.5 (0.1)	17.0 (0.1)
*p-*Coumarate	11.9 (0.2)	10.1 (0.3)*	10.6 (0.7)	12.8 (0.3)	12.2 (0.3)
Ferulate	5.2 (0.1)	4.7 (0.1)*	4.4 (0.1)*	4.6 (0.1)*	4.8 (0.1)*
Glucuronic acid	7.5 (0.1)	9.1 (0.2)*	8.6 (0.2)*	7.7 (0.1)	7.5 (0.1)
4-*O*-Methylglucuronic acid	4.4 (0.1)	4.5 (0.2)	3.8 (0.2)*	4.2 (0.1)	3.9 (0.1)*

Values are expressed in mg/g cell wall residue. Values in brackets are the SE from five biological replicates (*n* = 5 plants)

Asterisks (bold values) indicate significant differences from the wild type using the unpaired Student’s *t* test (*P < 0.05).

### Lignin degree of polymerization and monomeric composition

**Fig. 3:**
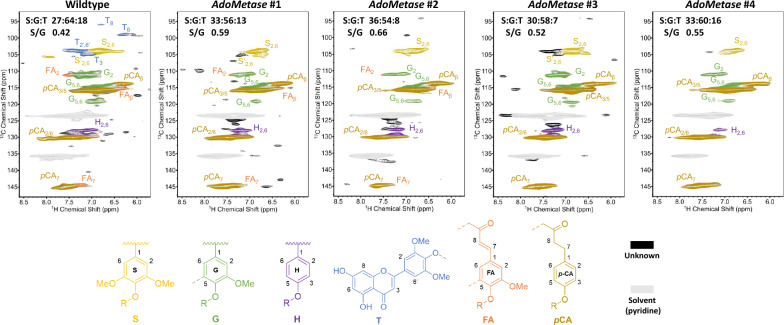
Lignin monome composition in wild-type and *AdoMetase* lines. Extractive-free stem and leaf biomass from fully mature plants was analyzed by 2D-HSQC NMR spectroscopy. Regions of partial short-range ^13^C–^1^H HSQC spectra are shown. For each line, the relative amount of lignin subunits including syringyl (S), guaiacyl (G), and tricin (T), as well as S/G values are provided. *p*CA: *p*-coumarate, FA: ferulate, H: *p*-hydroxyphenyl

**Fig. 4:**
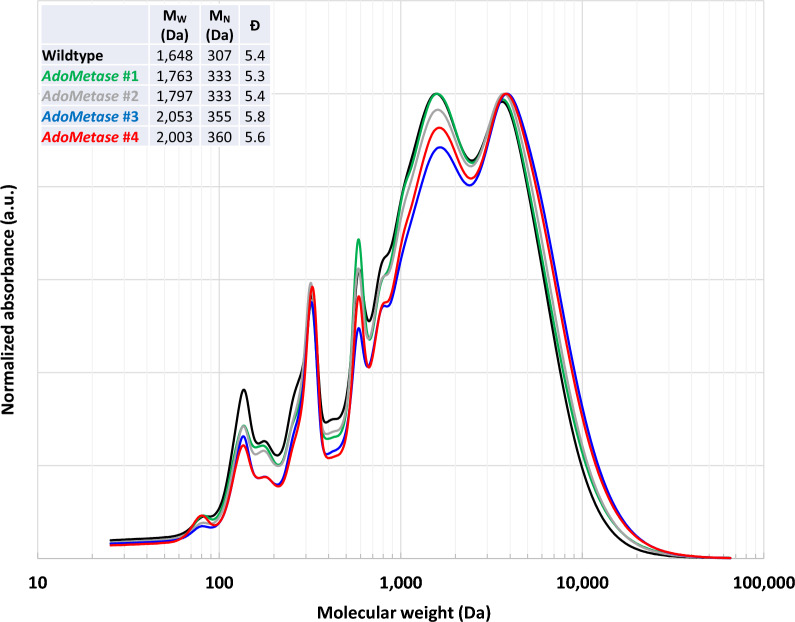
Gel-permeation chromatography (GPC) traces of solubilized lignin derived from wild-type and *AdoMetase* extractive-free biomass. Traces show the molecular weight distribution of lignin oligomers. For each line, lignin weight average molecular weight (M_W_), number average molecular weight (M_N_), and dispersity (Đ) are indicated

2D-HSQC NMR spectroscopy was used to analyze the monomeric composition of lignin (Fig. 3). The data showed a relative increase of S units and a decrease of G units in all four *AdoMetase* lines, which resulted in higher S/G ratios compared to the WT. A decrease of tricin was also observed in the transgenics. H units were not quantified due to the presence of overlapping signals that arise from protein contamination. The size distribution of lignin oligomers in cell walls was examined using gel permeation chromatography (Fig. 4). The distribution curves of the lignin molecular weights showed both decreased abundance in the lower molecular weight range (100–3000 Da) and increased abundance of higher molecular weight lignin (>3500 Da) in the case of the *AdoMetase* lines compared to WT. Accordingly, the estimated weight average molecular weight (M_W_) and number average molecular weight (M_N_) of lignin are both higher in transgenics, whereas lignin dispersities (Đ) remain similar to that of the WT.

### Saccharification potential

**Fig. 5:**
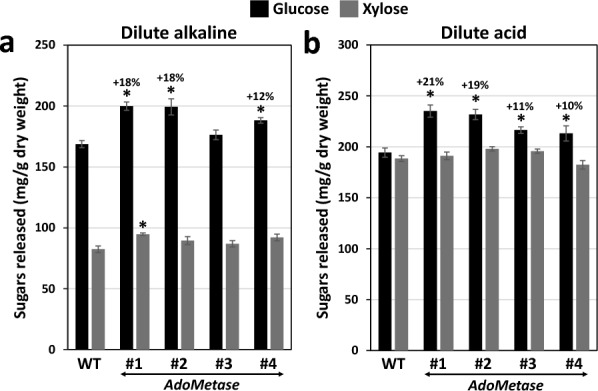
Biomass saccharification of the *AdoMetase* sorghum lines grown in the greenhouse. The amounts of glucose and xylose released after dilute alkaline (**a**) and dilute acid (**b**) pretreatments followed by 48 h of enzymatic digestion with an enzyme cocktail are shown. Values are means ± SE of five biological replicates (*n* = 5 plants). Asterisks indicate significant differences from the wild type (WT) using the unpaired Student’s *t* test (*P < 0.05)

The recalcitrance of engineered sorghum towards enzymatic degradation was evaluated by measuring the amount of sugars released from the biomass after dilute acid and mild alkaline pretreatments followed by a 48-h enzymatic hydrolysis using a mixture of cell wall degrading enzymes (Novozymes Cellic® CTec3). As shown in Fig. 5, higher amount of glucose was released from all *AdoMetase* lines. After alkaline pretreatment, glucose titers were ~18% higher in hydrolysates from the *AdoMetase* lines #1 and #2, and ~12% higher for *AdoMetase* #4. A significant increase in xylose (+15%) was also observed in the case of the *AdoMetase* #1 (Fig. 5a). After acid pretreatment, 10–21% more glucose was released from the biomass of transgenics compared to the WT (Fig. 5b). Considering that cell walls from both the control and transgenics contain similar amounts of glucose and xylose (Table 2), we conclude that the engineered lines have reduced cell wall recalcitrance and improved saccharification efficiency.

### Transcriptome analysis

**Fig. 6:**
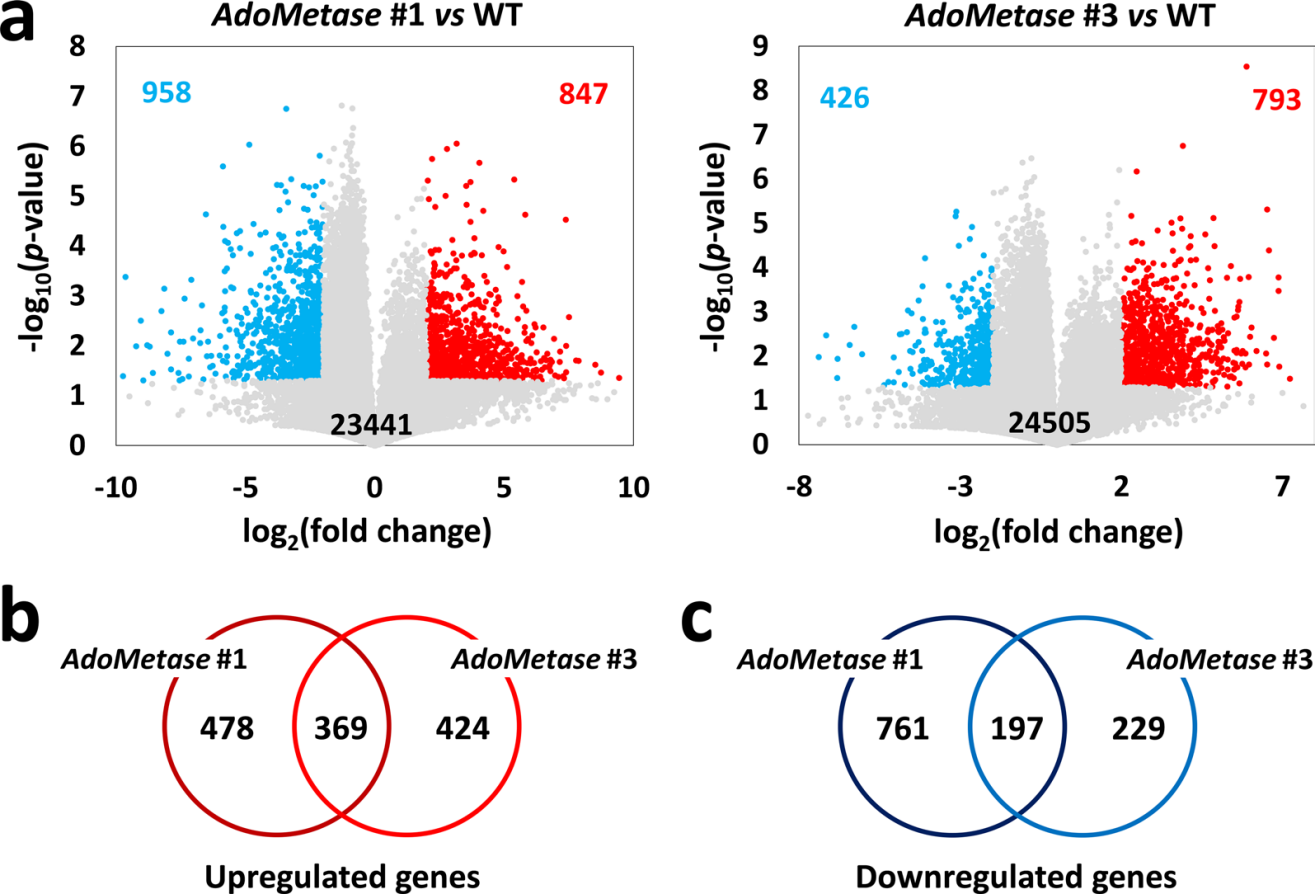
Analysis of DEGs in stems of the *AdoMetase* lines at the boot stage. **a** Volcano plots of transcripts identified in stems from WT and *AdoMetase* lines. The number of downregulated (in *blue*) and upregulated (in *red*) transcripts in *AdoMetase* #1 and *AdoMetase* #3 compared to WT control is indicated on each plot (log_2_-fold change +2/−2 and *P* value < 0.05). Grey dots represent transcripts that are not differentially expressed. Venn diagrams of upregulated (**b**) and downregulated (**c**) genes in *AdoMetase* #1 and #3

The impact of *AdoMetase* expression on the transcriptome was investigated by conducting RNA-seq analysis in stems of *AdoMetase* lines #1 and #3 at three developmental stages. In WT control plants, principal component analysis (PCA) demonstrated a clear distinction between stems analyzed at the three leaf, growing point differentiation (GPD), and boot stages (Additional File 1: Fig. S1a). A total of 28,734 transcripts were identified across all stages in WT, representing 89% of the 32,160 predicted protein-coding genes found in the sorghum genome. The expression level of these genes at the three stages is shown in Additional File 2: Dataset S1. Several transcripts were specific to the three leaf, GPD, and boot stage, while 25,334 genes were found to be expressed at all stages (Additional File 1: Fig. S1b). At each stage, principal component analysis of the transcripts allowed us to distinguish the different genotypes and showed a separation between *AdoMetase* #1 and #3, indicating alternate transcriptional changes between the two lines (Additional File 1: Fig. S2). The largest number of differentially expressed genes (DEGs) in transgenics compared to WT was observed at the boot stage, accounting for 958 upregulated and 847 downregulated genes in *AdoMetase* #1, and for 793 upregulated and 426 downregulated genes in *AdoMetase* #3 (Fig. 6a, Additional File 1: Fig. S3, Additional File 3: Dataset S2). Some overlap was found among DEGs in the two transgenic lines since 369 upregulated genes and 197 downregulated genes were common to both (Fig. 6b, c). However, only a limited number of genes were found consistently upregulated or downregulated at all three developmental stages in a given transgenic line (Additional File 1: Fig. S4). We performed GO and KEGG analyses to determine which biological processes, molecular functions, and metabolic pathways the DEGs identified in transgenics were significantly enriched in. The KEGG analysis revealed in the transgenics at the boot stage several DEGs associated with phenylpropanoid biosynthesis (i.e., 15 peroxidase genes), cytokinin synthesis, fatty acid elongation, cutin and suberin biosynthesis, and chromatin remodeling (Additional File 3: Dataset S2, Additional File 1: Fig. S5a). In particular, several genes associated with DNA metabolism encoding 19 different histone proteins, one histone demethylase, one histone methyltransferase, three DNA cytosine 5-methyltransferases, two DNA-3-methyladenine glycosylases, and one DNA repair protein RAD51 are overexpressed in *AdoMetase* #3 at the boot stage (yellow highlights in Additional File 3: Dataset S2). The GO analysis also indicated an enrichment of DEGs in biological processes related to cell wall organization, transmembrane transport, and stress and detoxification (Additional File 1: Fig. S5b).

### Metabolome analysis

**Fig. 7:**
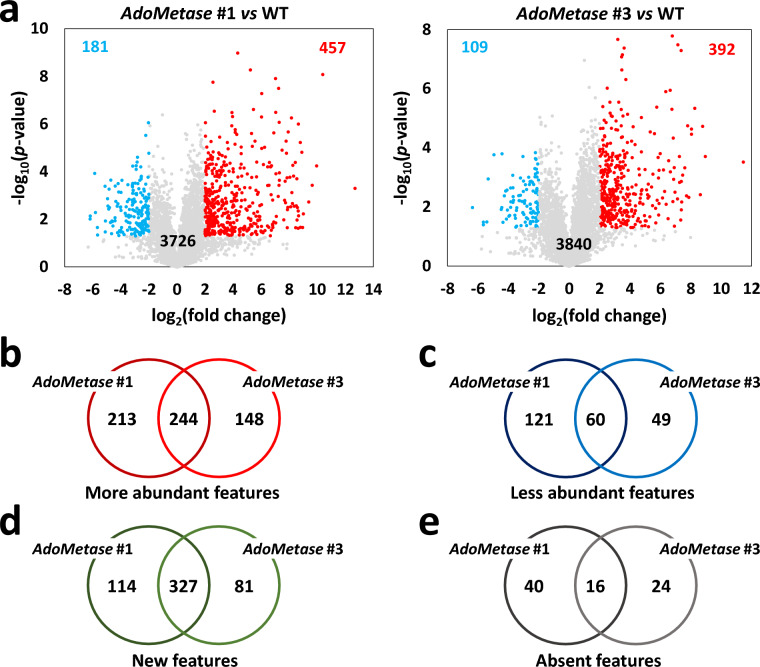
Analysis of differentially abundant features in stems of the *AdoMetase* lines at the boot stage. **a** Volcano plots of features detected in stems from WT and transgenic lines using HILIC chromatography (positive ionization mode). The number of decreased (in *blue*) and increased (in *red*) features in *AdoMetase* #1 and *AdoMetase* #3 compared to WT control is indicated on each plot (log_2_-fold change +2/−2 and *P* value < 0.05). Gray dots represent features that are not differentially abundant. Venn diagrams of features that are more abundant (**b**), less abundant (**c**), new (**d**), and absent (**e**) in *AdoMetase* #1 and #3

Separate aliquots of tissue samples used for transcriptome analysis were used for nontargeted metabolomics. We extracted metabolites with methanol and conducted metabolite profiling with a liquid chromatography–mass spectrometry platform that utilized gradient-based hydrophilic interaction (HILIC) both in the positive and negative ionization modes. The positive ionization allowed the detection of the largest number of unique features (5449) in the samples from WT plants, including 345, 147, and 173 features that are specific to the three leaf, GPD, and boot stage, respectively, whereas 3659 features were detected in all stages (Additional File 1: Fig. S6a, b). Principal component analysis (PCA) demonstrated a clear distinction between the three growth stages and between the different genotypes (Additional File 1: Fig. S6c, d, Fig. S7). Several features had different abundance in *AdoMetase* #1 and #3 compared to the WT. The highest number of differentially abundant features was found at the boot stage, representing 14.6% and 11.5% of the detected features in *AdoMetase* #1 and #3, respectively (Fig. 7a, Additional File 1: Fig. S8, Additional File 4: Dataset S3). At the boot stage, 244 more abundant features and 60 less abundant features were common in both transgenic lines compared to the WT (Fig. 7b, c). We also observed 327 features that are absent from both transgenic lines and detected 16 features that are new in both lines (Fig. 7d, e). Moreover, a majority of the differentially abundant features evidenced in the transgenic lines was only observed at a specific growth stage (Additional File 1: Fig. S9). Next, using GNPS, a subset of differentially abundant features observed in transgenics were classified and putatively identified: A majority of the differentially abundant metabolites identified in transgenics belong to the class of phenylpropanoids, alkaloids, and amino acids (Additional File 1: Fig. S10). As examples, the methylated flavonoids selgin, tricin, isorhamnetin and homoeriodictyol were decreased in the *AdoMetase* lines, whereas the AdoMetase product homoserine and a few derivatives such as threonine, Met, and isoleucine were significantly increased in the transgenics compared to WT (Additional File 1: Fig. S11). The content of the methylated flavone and tricin precursor chrysoeriol was unchanged in transgenics (data not shown), indicating that AdoMet levels are not limiting for its synthesis from luteolin.

### Field testing

The sorghum lines *AdoMetase* #1 and #3 were grown in the field until the soft dough stage in two different locations to evaluate their performance under natural environment. A composition analysis was performed on extractive-free cell wall residues obtained from stems and leaves. In *AdoMetase* #1, the content of acid-insoluble lignin was significantly reduced by 18% and 13% at the two different field sites, respectively, whereas line *Adometase* #3 showed no significant change (*P* < 0.05) (Table 3). For the Davis site, glucuronic acid content was increased by 43% and 16% in *AdoMetase* #1 and #3, respectively, whereas 4-*O*-methylglucuronic acid was reduced in *AdoMetase* #1 (8–27%) at both sites. As a result, the degree of glucuronic acid methylation was significantly reduced in both lines in the first site (*P* < 0.05). The amount of cell-wall-bound *p*-coumarate and ferulate in the transgenic lines was unchanged compared to the WT. Measurement of stover biomass showed yield reductions for both *AdoMetase* lines in the two locations (Additional File 1: Fig. S12). The yield penalty was more pronounced at the Davis site where the two lines were affected differently (69% reduction for *AdoMetase* #1 and 49% for *AdoMetase* #3) compared to the KARE site (~23% less biomass for both lines). In addition, the WT produced less biomass at the KARE site, possibly due to the sandy soil type and warmer temperatures at this location.

**Table 3:** Chemical composition of cell walls isolated from wild-type and *AdoMetase* lines grown in the field until the soft dough stage

	Davis site			KARE site		
	Wild type	*AdoMetase* #1	*AdoMetase* #3	Wild type	*AdoMetase* #1	*AdoMetase* #3
Glucose	302.6 (10.7)	298.1 (4.9)	286.1 (5.2)	327.7 (14.1)	336.0 (7.0)	332.6 (1.7)
Xylose	182.6 (15.2)	171.6 (4.2)	164.9 (7.8)	212.5 (1.9)	199.0 (3.3)*	202.4 (5.1)
Arabinose	25.6 (1.5)	31.3 (1.0)*	26.9 (1.1)	31.8 (2.2)	32.4 (1.8)	31.3 (0.9)
Acid-insoluble lignin	156.7 (10.6)	129.0 (1.3)*	139.3 (6.0)	145.6 (5.6)	126.5 (5.7)*	131.0 (4.4)
Acid-soluble lignin	16.2 (0.3)	17.5 (0.2)*	16.6 (0.1)	16.4 (0.1)	16.2 (0.1)	16.3 (0.2)
*p-*Coumarate	11.9 (2.1)	8.6 (0.8)	10.8 (0.6)	14.6 (0.9)	12.5 (1.6)	12.0 (0.9)
Ferulate	2.4 (0.5)	2.4 (0.1)	2.8 (0.2)	3.4 (0.1)	3.0 (0.4)	2.8 (0.3)
GlcA	22.3 (0.8)	31.9 (0.6)*	25.8 (0.6)*	24.2 (1.1)	26.9 (1.6)	25.7 (0.9)
4-*O*-MeGlcA	5.9 (0.4)	4.3 (0.2)*	4.2 (0.5)	6.1 (0.1)	5.6 (0.1)*	6.1 (0.2)

Values are expressed in mg/g cell wall residue. Values in brackets are the SE from four biological replicates (*n* = 4 plots). GlcA, glucuronic acid; 4-*O*-MeGlcA, 4-*O*-Methylglucuronic acid

For each site, asterisks (bold values) indicate significant differences from the wild type using the unpaired Student's *t* test (*P < 0.05)

**Fig. 8:**
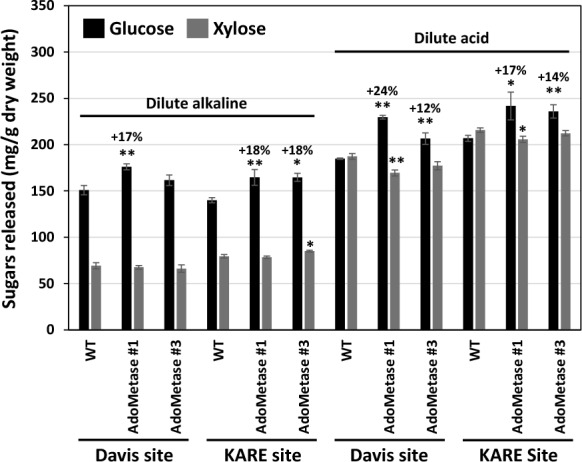
Biomass saccharification of the sorghum lines *AdoMetase* #1 and *AdoMetase* #3 grown in two different field sites in California. The data shows the amounts of glucose and xylose released after dilute alkaline and dilute acid pretreatments followed by 48 h of enzymatic digestion with an enzyme cocktail. Values are means ± SE of four biological replicates (*n* = 4 plots). For each site and pretreatment method, asterisks indicate significant differences from the wild type (WT) using the unpaired Student’s *t* test (*P < 0.1, **P < 0.05)

Saccharification assays were conducted on the biomass from stems and leaves. As previously observed in the greenhouse experiment, the two engineered sorghum lines showed improvements of saccharification efficiency, releasing up to 18% and 24% more glucose after dilute alkaline and dilute acid pretreatments, respectively (Fig. 8).

## Discussion

In sorghum, genetic mutations that introduce premature stop codons in a gene encoding a COMT (*Sobic.007G047300*) lead to brown midrib (bmr) phenotypes due to changes in lignin composition [26]. For example, biomass from the well-studied *bmr12* COMT mutant contains ~6% less acid-insoluble lignin and shows a decrease in the lignin S/G ratio and tricin content, resulting in slight improvements of sugar yields (<10%) after dilute acid or mild alkali biomass pretreatments followed by enzymatic saccharification [14, 27, 28]. These observations also suggest that other COMTs besides Sobic.007G047300 are involved in lignin biosynthesis in sorghum since S and T units remain present in the *bmr12* mutant. In the current study, the heterologous expression of AdoMetase in sorghum reduces AdoMet content and potentially affects the activity of any OMTs that participate in lignin synthesis, resulting in up to 18–19% reductions of acid-insoluble lignin in the greenhouse and the field. The *AdoMetase* sorghum lines show delayed flowering, as previously observed in *bmr12* mutants, but they also show reductions of plant height and biomass yields, which is in contrast with *bmr12* plants [29–32]. Such a short phenotype was previously observed in the sorghum *bmr19* mutant that is presumably affected in AdoMet synthesis due to a mutation in a FPGS gene [33]. Thus, by using the *pSbCOMT* promoter to drive *AdoMetase* expression, our results suggest that not only COMT but also CCoAOMTs are impacted in their activity in the *AdoMetase* lines. First, unlike the *bmr12* mutants that have reduced lignin S/G ratios, the *AdoMetase* lines at maturity show an increased S/G, as previously observed in the *bmr19* mutant and maize plants silenced in a CCoAOMT gene or carrying mutations in FPGS (*bm4*) or MTHFR (*bm2*) [19, 22, 34, 35]. Arguably, it is not excluded that *AdoMetase* expression declines at later stages during the reproductive growth phase, which would marginally affect lignin biosynthesis in maturing stems that typically produce S-rich lignin, and result in lignin with higher S/G ratio as observed in fully mature transgenic plants [36]. Second, an increase of ferulate and a decrease of *p*-coumarate bound to the cell wall were measured in *bmr12* mutants [28], which is in contrast with our *AdoMetase* lines that show no change in cell-wall-bound *p*-coumarate and a reduction of ferulate at maturity (Table 2). The lignin in the *AdoMetase* lines shows increased M_W_ and M_N_, which is different from the data usually observed in engineered plants that have higher lignin S/G [37]. This difference is possibly due to the reduction of T units since M_W_ and M_N_ were shown to be negatively correlated with tricin content in isolated lignin fractions, consistent with the exclusive occurrence of tricin as pendant end-groups in lignin and its putative role as nucleation sites for lignin polymerization [5, 38].

Based on our RNA-seq data, we did not find any significant changes in the expression of known genes (i.e. *Bmr2*, *Bmr6*, *Bmr12*, *Bmr19*, *Bmr30*, and *SbMYB60*) or putative genes involved in lignin biosynthesis in the stems of the *AdoMetase* lines at the three different growth stages (Additional File 3: Dataset S2, log_2_-fold change +2/−2 and *P* < 0.05) [18, 26, 39–43]. Moreover, none of the seven CCoAOMT genes found in the sorghum genome showed differential expression in transgenics compared to the WT [44]. We found that 29 out of the 40 OMT genes present in the sorghum genome are expressed in stems (Additional File 2: Dataset S1) [45]. Among those, only two genes are significantly downregulated in both *AdoMetase* lines: *Sobic.007G170500* (three-leaf stage) and *Sobic.002G079500* (boot stage) (Additional File 3: Dataset S2). *Bmr12* (*Sobic.007G047300*) is the OMT gene with the highest expression level, showing DESeq2 normalized values of 23,525, 26,202.5, 33,304 at the three leaf, GPD, and boot stage, respectively, followed by *Sobic.009G197000* (i.e. DESeq2 values of 8,296, 11,474, and 18,576 at the three above-mentioned stages). The 27 other OMT genes show much lower expression levels in the stem (i.e. DESeq2 values <645). Overall, this data indicates that the reduction of lignin measured in the *AdoMetase* lines is probably the result of lower cytosolic AdoMet pools rather than downregulation of the genes involved in lignin biosynthesis. Moreover, the reduction of the degree of glucuronic acid methylation on xylan indicates a diminution of the AdoMet pool inside the Golgi where the corresponding glucuronic acid methyltransferases are located [46].

The RNA-seq data indicates that biological processes other than lignification and xylan methylation may be impacted in the *AdoMetase* lines, which is perhaps not surprising considering the ubiquitous role of the AdoMet cofactor. Notably, the expression of *AdoMetase* modifies the expression of several genes encoding histone and DNA base methyltransferases, as wells as histone proteins. These changes in the expression of enzymes involved in DNA metabolism could result in epigenetic modifications that affect gene expression more broadly, and explain the delay in growth and development observed in the *AdoMetase* lines under field conditions [47]. Changes in the expression of genes involved in the metabolism of the cytokinin zeatin, which derives from the nucleobase adenine, also suggest some possible perturbations of hormone signaling in the *AdoMetase* lines. For example, one of the genes encoding 1-aminocyclopropane-1-carboxylate synthase (*Sobic.001G105900*), an ethylene biosynthetic enzyme that uses AdoMet as cofactor, is upregulated in both *AdoMetase* lines at the three-leaf stage, presumably as part of a compensation mechanism (Additional File 3: Data S2). In addition, the overexpression of multiple peroxidase genes in transgenics is indicative of a stress response due to higher levels of reactive oxygen species.

At the metabolite level, several compounds belonging to the class of phenylpropanoids and alkaloids are differentially abundant in the *AdoMetase* lines. *O*-methyltransferases are indeed involved in the methylation of a wide range of secondary metabolites such as alkaloids and aromatics, so that reductions in AdoMet pools are expected to impact their relative amounts [48]. For example, we showed that two flavones synthesized by SbCOMT/Bmr12 (selgin and tricin) are reduced in metabolite extracts from the *AdoMetase* lines compared to wild-type plants. Other unknown *O*-methyltransferases involved in the synthesis of methylated compounds such as isorhamnetin and homoeriodictyol are probably affected by a reduction of AdoMet since these two flavonoids are also reduced in transgenics (Additional File 1: Fig. S11a). The compound class ‘amino acids’ is also represented in the differentially abundant metabolites identified in the *AdoMetase* lines. In particular, the AdoMetase product homoserine appears to be partially recycled into threonine, isoleucine, and Met, which are all increased in transgenics (Additional File 1: Fig. S11b). The increase in Met could represent a cellular response to compensate for the reduction of AdoMet pools since Met is the AdoMet precursor. Met accumulation may also occur from the recycling of 5ʹ-methylthioadenosine (i.e. the other AdoMetase product) via the Yang cycle because the early intermediate 5-methylthioribose of this cycle is increased in the transgenic lines (Additional File 1: Fig. S11b). A significant increase of *S*-adenosylhomocysteine (AdoCys) was also observed (Additional File 1: Fig. S11b), which is somehow unexpected since AdoCys is a by-product of AdoMet-dependent methyltransferases that usually undergoes hydrolysis into adenosine and homocysteine by AdoCys hydrolase. Whether higher AdoCys content is the consequence of AdoCys hydrolase reverse reaction or another unknown enzymatic activity in transgenics remains to be determined [49, 50]. Interestingly, augmenting AdoCys levels was achieved in switchgrass by silencing AdoCys hydrolase, which successfully led to lignin reductions, supposedly via inhibition of COMTs/CCoAOMTs by AdoCys [51].

## Conclusions

This study demonstrates the effectiveness of expressing *AdoMetase* in sorghum to reduce lignin and improve enzymatic conversion of biomass to simple sugars. Our work also highlights in transgenic lines some pleiotropic effects on metabolic pathways that require AdoMet for methylation reactions. Thus, fine-tuning *AdoMetase* expression by using promoters active at later developmental stages should be evaluated in order to mitigate adverse effects on plant growth. For example, a promoter that is induced in response to shorter day length could be appropriate in the case of late planting during the growing season. Moreover, introducing *AdoMetase* in energy sorghum varieties could represent a viable approach to reduce biomass recalcitrance and maintain high biomass yields [52].

## Materials and methods

### Plant growth conditions

Plants were grown at the UC Berkeley greenhouse Oxford facility with minimum temperature set at 22 °C. Seeds were germinated directly on soil (Sunshine mix #4, Sun Gro) in one-gallon pots and plants were grown until seeds reached the black layer stage (i.e. full physiological maturity). One tablespoon of Osmocote Plus 15-9-12 was added to the soil biweekly until the flowering stage. Watering was stopped at the end of the growing period and pots containing plants were allowed to dry for another 3 weeks. Plants without their panicles were harvested, further dried in an oven at 50 °C for 5 days, and subsequently ground using a knife mill equipped with a 1-mm mesh (Model 4 Wiley Mill, Thomas Scientific). For cell wall composition analyses, samples were additionally ground into a fine powder using a ball mill (Mixer Mill MM 400, Retsch Inc.) with stainless-steel balls.

### Design of the *pSbCOMT:AdoMetase* construct

The promoter sequence of the *SbCOMT* gene (1990 bp) was amplified by PCR using genomic DNA from *Sorghum bicolor* (variety BTx623) and the primers listed in Additional File 1: Table S1. The fragment was cloned into the pBca9145 vector [53] via In-Fusion cloning (Takara Bio USA) to generate a level-0 construct. A DNA sequence encoding AdoMetase from the enterobacteria phage T3 (GenBank: CAA28477.1) was codon-optimized for expression in sorghum and synthesized as a gene fragment by GenScript. The sequence contained flanking BsaI restriction sites plus extra homologous sequences for cloning into pBca9145. The *pSbCOMT:AdoMetase* construct was obtained using the jStack cloning method [53]. The corresponding level-0 and level-1 intermediate plasmids are listed in Additional File 1: Table S2. Plasmid sequences are available at the Inventory of Composable Elements source registry (http://public-registry.jbei.org).

### Sorghum transformation and genotyping

The *Agrobacterium tumefaciens* EHA105 strain was used to transform sorghum (*S. bicolor*, variety Wheatland) using immature embryos as previously described [54]. TaqMan Real-time PCR assays (Thermo Fisher Scientific) were performed on gDNA isolated from primary transformants using primers specific to the *nptII* and *AdoMetase* gene sequences to identify transformants with a single-copy event. Plants homozygous for the transgene and WT segregants were identified using the same approach for four selected lines in the T1 generation. T2 seeds from one T1 WT segregant plant from each line were pooled and used as WT controls. Plants in the T3 generation were used for all greenhouse experiments.

### Analysis of *AdoMetase* expression

*AdoMetase* expression was assessed using reverse transcription quantitative PCR (RT-qPCR). Total RNAs were extracted from stems of 3-week-old plants using the RNeasy Plant Mini Kit (Qiagen) and first-strand cDNA synthesis was conducted using the SuperScript IV First-Strand Synthesis kit (Thermo Fisher Scientific) as previously described [55]. qPCR on first-strand cDNA was performed using 35 cycles consisting of 5 s at 95 °C for denaturation and 15 s at 60 °C for annealing and amplification. The relative quantification of *AdoMetase* transcripts was calculated using the 2^−ΔCT^ method and normalized to the reference gene *PP2A* [56]. The results are the average from four biological replicates. RT-qPCR primers are listed in Additional File 1: Table S1.

### RNA-seq analysis

Four biological replicates from two *AdoMetase* lines (#1 and #3) and WT control were used for large-scale transcriptomic analysis. Entire stems from seedlings (three-leaf stage) and 3-cm stem sections collected 5 cm from the soil surface (growing point differentiation and boot stages) were flash-frozen in liquid nitrogen and stored at −80 °C until processing. Frozen samples were pulverized using a freezer mill (model 6875D, SPEX SamplePrep LLC). Each sample was ground twice for 1 min at a grinding rate of 15 counts per sec, including a 15 s break between the two cycles. Total RNA was extracted using the RNeasy Plant Mini Kit (Qiagen) and treated with RNAse-free DNAse. RNA sequencing, RNA-seq read filtering, and raw gene counts was done as described previously [57]. Filtered reads from each library were aligned to the *Sorghum bicolor v5.1* reference genome (https://phytozome-next.jgi.doe.gov/info/Sbicolor_v5_1) using HISAT2 version 2.2.1 [58].

### Metabolite analysis

To quantify AdoMet, 5ʹ-methylthioadenosine, homoserine, threonine, isoleucine, AdoCys, methionine, and 5-methylthioribose, metabolites were extracted from 3-week-old plants using 5% (w/v) trichloroacetic acid and analyzed using high-performance liquid chromatography (HPLC), electrospray ionization (ESI), and time-of-flight (TOF) mass spectrometry (MS) as previously described [24]. Flavonoids were extracted from stems and leaves of fully mature plants and quantified after acid-hydrolysis of the metabolite extracts using the LC–MS method described in [14]. Measurement of the metabolites was performed via the analysis of standard compounds using the same LC–MS methods. 5-Methylthioribose was identified based on accurate mass measurements of LC–MS peaks ([M + H]^+^ = 181.0529 at <3.5 ppm) and CID–MS/MS fragmentation patterns showing major [M + H]^+^ ions at 57.0340, 71.0491, 85.0284, 117.0546, 121.0318, 149.0267, and 163.0423 *m*/*z* using an collision energy of 10 eV.

Aliquots of the homogenized biomass samples used for RNA-seq analysis were used for untargeted metabolomics. To obtain metabolite extracts, frozen tissue powder was weighed and mixed with methanol at a ratio of 100 µL of solvent per mg of powder. Samples were vortexed for 1 min, then incubated at room temperature for 20 min with continuous mixing, centrifuged at 20,000×*g* for 5 min and the supernatant filtered through 0.45-µm PTFE filters. Three extraction control samples were obtained by following the same procedure, but without any tissue powder. Filtered metabolite extracts and controls were vacuum-dried and resuspended in 100% methanol containing an internal standard mix of isotopically labeled compounds for UHPLC–MS/MS normal phase chromatography using a HILIC column (InfinityLab Poroshell 120 HILIC-Z, 2.1 × 150 mm, 2.7 µm, Agilent) and following the method described in [57]. The raw data from the metabolite LC–MS runs is provided in Additional File 5: Dataset S4. For data analysis, features with 0.8 min < RT < 18.5 min and max peak height fold-change between sample and extraction control >3 were selected. LC–MS data was analyzed using custom Python code [59], with each detected peak assigned a level of confidence indicated by a score from 0 to 3 in the compound identification. We performed a Feature-Based Molecular Networking workflow using MZmine 2 [60] and Global Natural Products Social Molecular Networking (GNPS; http://gnps.ucsd.edu) [61]. The MZmine workflow was used to generate a list of features obtained from extracted ion chromatograms containing chromatographic peaks within a narrow *m*/*z* range and filtered to remove isotopes. For each feature, the most intense fragmentation spectrum was uploaded to GNPS for putative identification by comparison with mass spectra deposited in the database. Compound classes are attributed to identified compounds using ClassyFire [62] or NPClassifier [63].

### Cell wall composition analysis

Ball-milled biomass (1 g) was sequentially extracted using a Dionex ASE 350 accelerated solvent extractor set to 7 min static extraction cycles (Thermo Fisher Scientific). Solvents (5 mL) were water (two cycles), 80% (v/v) ethanol–water (ten cycles), 50% (v/v) methanol–chloroform (one cycle), and acetone (one cycle). Acid-insoluble lignin, acid-soluble lignin (absorbance at 320 nm), cell wall monosaccharides (glucose, xylose, and arabinose), and cell-wall-bound aromatics (*p*-coumarate and ferulate) were measured as previously described [64, 65]. Glucuronic acid and 4-*O*-methylglucuronic acid were quantified using HPLC–ESI–qTOF–MS analysis and a BioRad Fermentation Monitoring HPX-87H 150 × 7.8 mm column. The mobile phase was 0.1% formic acid in water at a flow rate of 0.50 mL/min and a total run time of 13 min. Other parameters were as follows: Column temperature: 50 °C, maximum pressure: 100 bar, ionization mode: negative, gas temperature: 340 °C, drying gas flow: 11 L/min, nebulizer: 30 psig, fragmentor: 140 V, skimmer: 50 V, octopole radio frequency voltage: 195 V, VCap; 3500 V, acquisition range (*m*/*z*): 50–700, acquisition rate (spectra/s): 0.86.

### 2D-HSQC NMR

For each sorghum line, the sample consisted of a pool of equal amount of extractive-free cell wall residue from five plants. Preparation of the samples for 2D-HSQC NMR experiments was described previously [66, 67]. In a 5-mm NMR tube, ~ 100 mg of extracted biomass was added along with 1 ml of pre-mixed DMSO–*d*_6_/pyridine–*d*_5_ solvent (4:1, v/v) to form a gel. The NMR tubes were sealed and sonicated for 4 h with a 30 min interval every hour until the gel became apparently homogeneous. The 2D-HSQC NMR spectra were collected on a Bruker Avance I 800 MHz spectrometer equipped with a Bruker Triple Resonance Probe (TXI) at 310 K. A standard Bruker pulse sequence (hsqcetgpsisp2.2) was used with parameters that are typical for lignocellulose samples. Data were acquired using Bruker’s TopSpin software (version 4.1.0). The HSQC spectra were collected from 11 to −1 ppm in the *F*_2_ (^1^H) dimension with 1,024 data points and 53 ms acquisition time with an interscan pulse delay of 1 s, and from 165 to −10 ppm in the *F*_1_ (^13^C) dimension with 256 data points and 3.5 ms acquisition time. For each evolution period (t1) increment, 256 scans were recorded. The central DMSO solvent peak was used as a reference for the chemical shift calibration for all samples (*δ*_C_ 39.5 ppm, *δ*_H_ 2.5 ppm). All HSQC spectra were processed using typical cosine-squared apodization in both *F*_2_ and *F*_1_ dimensions, and the contours were integrated using MestreNOVA (version 14, Mestrelab Research). Peaks were assigned according to published data [66–69].

### Gel permeation chromatography (GPC)

Pools of extracted biomass from five plants were used for each line. Samples were incubated in 2.5 mL of acetic acid and acetyl bromide (92:8) and stirred at 50 °C for 2 h to dissolve the lignin [70]. Acetylated lignin was dissolved in tetrahydrofuran and filtered through 0.2-µm PTFE filters. GPC analysis of biomass-derived lignin fragments was conducted using an Ecosec HLC-8320GPC (Tosoh Bioscience) equipped with Agilent Technologies PLgel 5 μm Mixed-D column and a diode array detector. Tetrahydrofuran, spiked with 250 ppm of butylated hydroxytoluene, was used as the mobile phase with a flow rate of 1 ml min^−1^ and a column temperature of 40 °C. Polystyrene oligomers ranging from 162 to 29,150 g mol^−1^ (Agilent Technologies #PL2013) were dissolved in tetrahydrofuran and used as standards for calibration using the conditions described above.

### Saccharification

The biomass from leaves and stems (panicles removed) was used for saccharification assays. Dilute acid (1.2% w/v sulfuric acid) and alkali (0.25% w/v sodium hydroxide) were used for biomass pretreatments. The enzyme mixture Ctec3 (Novozymes) was used for polysaccharide hydrolysis. The procedure and measurement of released monosaccharides were performed as previously described [71, 72].

### Field trial

Seeds (T4 generation) from *AdoMetase* #1, *AdoMetase* #3, and pooled wild-type segregants were planted in two field trials conducted at the University of California Davis Plant Sciences Research Farm (‘Davis site’, Davis, CA) and the Kearney Agricultural Research and Extension Centre (‘KARE site’, Parlier, CA) in 2023 under a USDA–APHIS Biotechnology Regulatory Services permit for regulated sorghum (BRS# 124-Q89IS3R, AUTH# 241864) as previously described [73, 74]. The Davis site has a Yolo clay loam soil (fine-silty, mixed thermic Fluventic Haploxerept) and the KARE site has a Hanford sandy loam soil. Seeds were planted on June 22, 2023. Percent germination was taken for each line, and adjusted for a target seeding rate of 197,600 seeds ha^−1^. The experiment was a randomized complete block design with four replicates. Row spacing was 0.76 m. Plots measured 3 × 6 m and comprised two rows. A commercial sorghum variety (NK8828 from S&W Seed Company) was planted as border rows to limit edge effects. Before planting, the amount of N–P–K fertilizer applied at the Davis site was 224, 91, and 22 kg ha^−1^, respectively. At the KARE site, the amount of N–P–K was 115, 58, and 233 kg ha^−1^, respectively, with an additional 77 kg ha^−1^ of in-season nitrogen from a urea source injected into the drip system. Sivanto was applied at a rate of 481 g ha^−1^ on August 7 in the KARE site for sugarcane aphid control. Irrigation water was applied utilizing surface furrow methods at the Davis site and drip irrigation at the KARE site to satisfy the fully watered evapotranspiration requirement for sorghum during the growing season. Evapotranspiration was estimated from nearby weather stations. All panicles on each plant were covered with pollinating bags prior to anthesis to prevent pollen flow according to APHIS permit regulations. Plot harvest took place on October 27, 2023. Panicles (heads with immature grain) were removed by hand. Remaining stover was harvested using a Wintersteiger Cibus forage chopper (Wintersteiger). Subsamples of approximately 500 g of fresh weight stover material were taken from each harvested plot, and dried at 55 °C for 1 week in a forced air oven to determine dry matter and calculate yield. For composition analyses and saccharification assays, stover samples were first milled using a Model 4 Wiley Mill equipped with a 1-mm mesh (Thomas Scientific) and further ground into powder as previously described.

### Data analysis

Principal component analysis plots were obtained with MetaboAnalyst 5.0 (https://www.metaboanalyst.ca/) and Venn diagrams were generated with the webtool available at https://bioinformatics.psb.ugent.be/webtools/Venn/. For the Gene Ontology (GO) enrichment analysis, the web-based g:Profiler tool was used to identify overrepresented biological processes in the *AdoMetase* lines [75]. The build-in algorithms of g:Profiler was used to correct for multiple testing and to identify the leading GO terms and remove the redundant terms in each function component. Leading GO terms that were significantly enriched (adjusted *P* value ≤0.05) in at least one *AdoMetase* line were visualized in a dot plot using the ggplot2 package v3.4.4 in R v4.3.1 [76, 77]. For the KEGG analysis, *enrichKEGG* function with the clusterProfiler package v4.8.3 [78] in R was applied to identified overrepresented KEGG categories in the *AdoMetase* lines based on the UniProt IDs of their differentially expressed genes. The *P* value was adjusted using FDR method with a cutoff of 0.1. The significantly enriched KEGG categories were visualized in a dot plot as described above.

## Supplementary Information

Additional file 1.

Additional file 2.

Additional file 3.

Additional file 4.

## Data Availability

All the data supporting the conclusions of this article is included within the article and its additional files. The RNA-seq raw data sets in this study are available at the NCBI Sequence Read Archive under BioProject IDs PRJNA1098655–PRJNA1098666 and PRJNA1098671–PRJNA1098695. The raw metabolite data can be accessed at the MassIVE public depository under the data set identifier MSV000094762.

## Abbreviations

AdoCys: *S*-Adenosylhomocysteine
AdoMet: *S*-Adenosylmethionine
AdoMetase: S-adenosylmethionine hydrolase
bmr: Brown midrib
CCoAOMT: Caffeoyl-CoA *O*-methyltransferase
COMT: Caffeic acid *O*-methyltransferase
FPGS: Folylpolyglutamate synthase
GPC: Gel-permeation chromatography
GPD: Growing point differentiation
MTHFR: 5-Methyltetrahydrofolate reductase
PCA: Principal component analysis
