# Supplementary material for: Engineered reduction of S-adenosylmethionine alters lignin in sorghum
Source: Biotechnol Biofuels Bioprod. 2024 Oct 15;17:128. doi: 10.1186/s13068-024-02572-8 (PMC11481400; doi:10.1186/s13068-024-02572-8)
Supplement: Supplementary file 1 — Additional file 1. [file 13068_2024_2572_MOESM1_ESM.pdf]

Article title: **Engineered reduction of *S*-adenosylmethionine alters lignin in sorghum**

Authors: Yang Tian, Yu Gao, Halbay Turumtay, Emine Akyuz Turumtay, Yen Ning Chai, Hemant Choudhary, Joon-Hyun Park, Chuan-Yin Wu, Christopher M. De Ben, Jutta Dalton, Katherine B. Louie, Thomas Harwood, Dylan Chin, Khanh M. Vuu, Benjamin P. Bowen, Patrick M. Shih, Edward E. K. Baidoo, Trent R. Northen, Blake A. Simmons, Robert Hutmacher, Jackie Atim, Daniel H. Putnam, Corinne D. Scown, Jenny C. Mortimer, Henrik V. Scheller, Aymerick Eudes

Additional File 1

Table S1: List of plasmids used in this study
Table S2: List of primers used in this study
Figure S1: Analysis of transcripts isolated from stems of WT sorghum
Figure S2: PCA plots of the transcripts identified in stems of WT and *AdoMetase* lines #1 and #3 at three developmental stages
Figure S3: Volcano plots of transcripts identified in stems from WT and *AdoMetase* lines at the three-leaf and growing point differentiation (GPD) stages
Figure S4: Venn diagrams of DEGs at the three developmental stages for each *AdoMetase* line
Figure S5: Dot plots of KEGG and GO enrichment analyses of DEGs identified in *AdoMetase* #1 and #3 at three developmental stages
Figure S6: Venn diagrams and PCA plots of the features detected in stems of WT sorghum at three different growth stage using HILIC chromatography (positive ionization mode)
Figure S7: PCA plots of the features detected in stems of WT and *AdoMetase* lines #1 and #3 at three developmental stages using HILIC chromatography (positive ionization mode)
Figure S8: Volcano plots of features detected in stems from WT and transgenic lines at the three-leaf and GPD stages using HILIC chromatography (positive ionization mode)
Figure S9: Venn diagrams of the differentially abundant features observed in the *AdoMetase* lines at three different growth stage using HILIC chromatography (positive ionization mode)
Figure S10: Classification of a subset of differentially abundant metabolites identified in stems of the *AdoMetase* lines at three developmental stages using HILIC chromatography (positive ionization mode)
Figure S11: Relative content of targeted metabolites in the *AdoMetase* lines
Figure S12: Stover biomass yields from the WT and *AdoMetase* lines grown until the soft dough stage in two different field sites in California.

Table S1. List of plasmids used in this study.

Construct name	Level	Backbone	Description	JBEI ICE ID
pSbCOMT:AdoMetase	2	pPMS074	*tOCS-Kan^R-pSbCOMT:AdoMetase-tNOS*	JBx_090071
pSbCOMT:AdoMetase-Lv1	1	pPMS028	Level-1 construct obtained with level-0 parts: {L_tOCS-Kan^R}, {P_SbCOMT}, {AdoMetase}, and {T_tNOS}	JBx_238485
{L_tOCS-Kan^R}	0	pBca9145	*Agrobacterium* octopine synthase terminator and plant kanamycin selectable marker, primary linker	JBx_065723
{P_SbCOMT}	0		2-kb promoter region of the COMT gene (*BMR12*) from sorghum (GenBank: CM000766.3)	JBx_090061
{AdoMetase}	0		AdoMetase gene from enterobacteria phage T3 (GenBank: CAA28477.1), codon-optimized.	JBx_090023
{T_tNOS}	0		*Agrobacterium* nopaline synthase terminator	JBx_042266

Table S2. Primers used in this study.

**Primer name**	**Purpose/ Target**	**Sequence (5'-3')**
pSbCOMT-Fw	*pSbCOMT* / gDNA	CGCTAAGGATGATTTCTGGAATTCGGTCTCTGGAGGCATGAAGTGCTGACGTG
pSbCOMT-Rv		CAGCTCGAGTTAGGATCCGGTCTCACATTGGCTAGCTAGCTAGCTGACG
AdoMetase-qPCR-Fw	RT-qPCR / *AdoMetase* cDNA	GCCTGTAATGAGTGGGAACA
AdoMetase-qPCR-Rv		AGACGTTCGGCCTTGTAAC
PP2A-qPCR-Fw	RT-qPCR / *PP2A* cDNA	AACCCGCAAAACCCCAGACTA
PP2A-qPCR-Rv		TACAGGTCGGGCTCATGGAAC

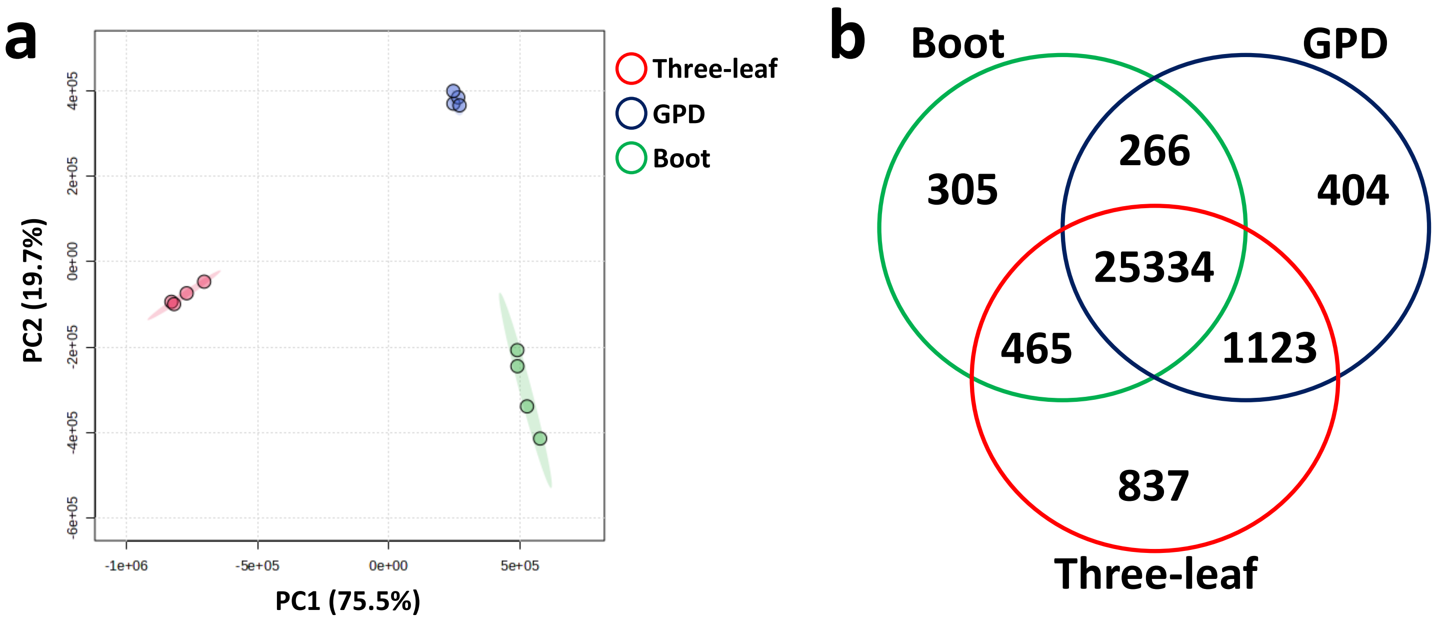

Figure S1: Analysis of transcripts isolated from stems of WT sorghum. PCA plot (**a**) and venn diagram (**b**) of the 28,734 unique transcripts identified at different developmental stages. GPD, growing point differentiation.

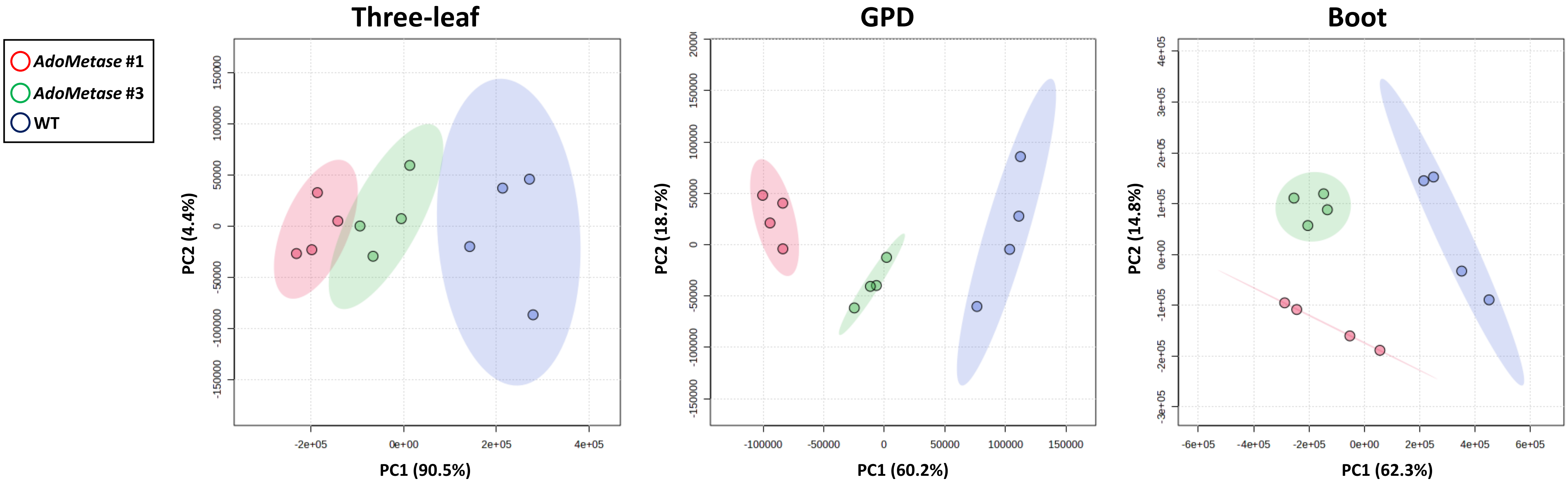

Figure S2: PCA plots of the transcripts identified in stems of WT and *AdoMetase* lines #1 and #3 at three developmental stages.

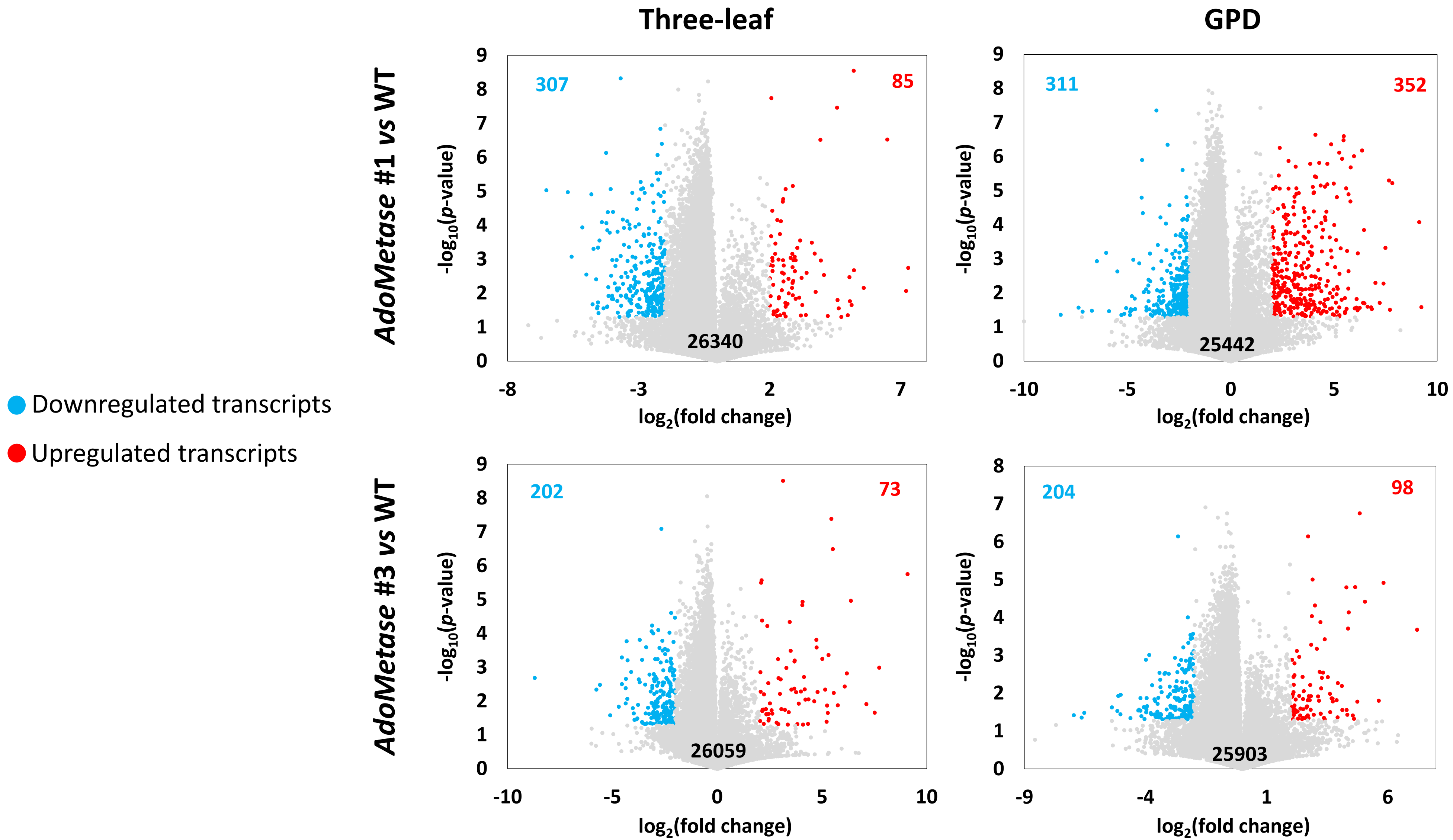

Figure S3: Volcano plots of transcripts identified in stems from WT and *AdoMetase* lines at the three-leaf and growing point differentiation (GPD) stages. The number of downregulated (in blue) and upregulated (in red) transcripts in *AdoMetase* #1 and *AdoMetase* #3 compared to WT control is indicated on each plot (log_2-fold change +2/-2 and *p*-value < 0.05). Grey dots represent transcripts that are not differentially expressed.

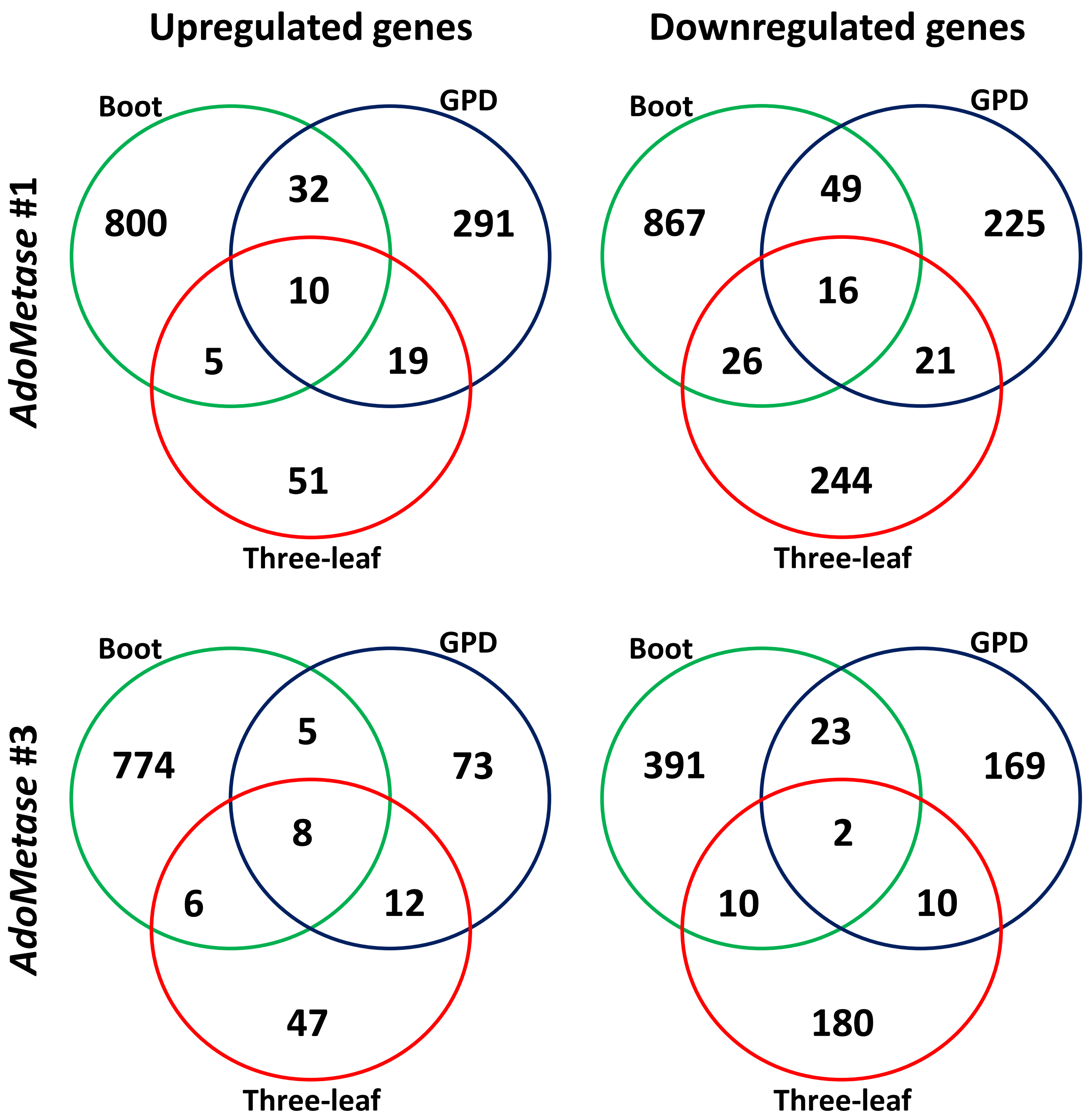

Figure S4: Venn diagrams of DEGs at the three developmental stages for each *AdoMetase* line.

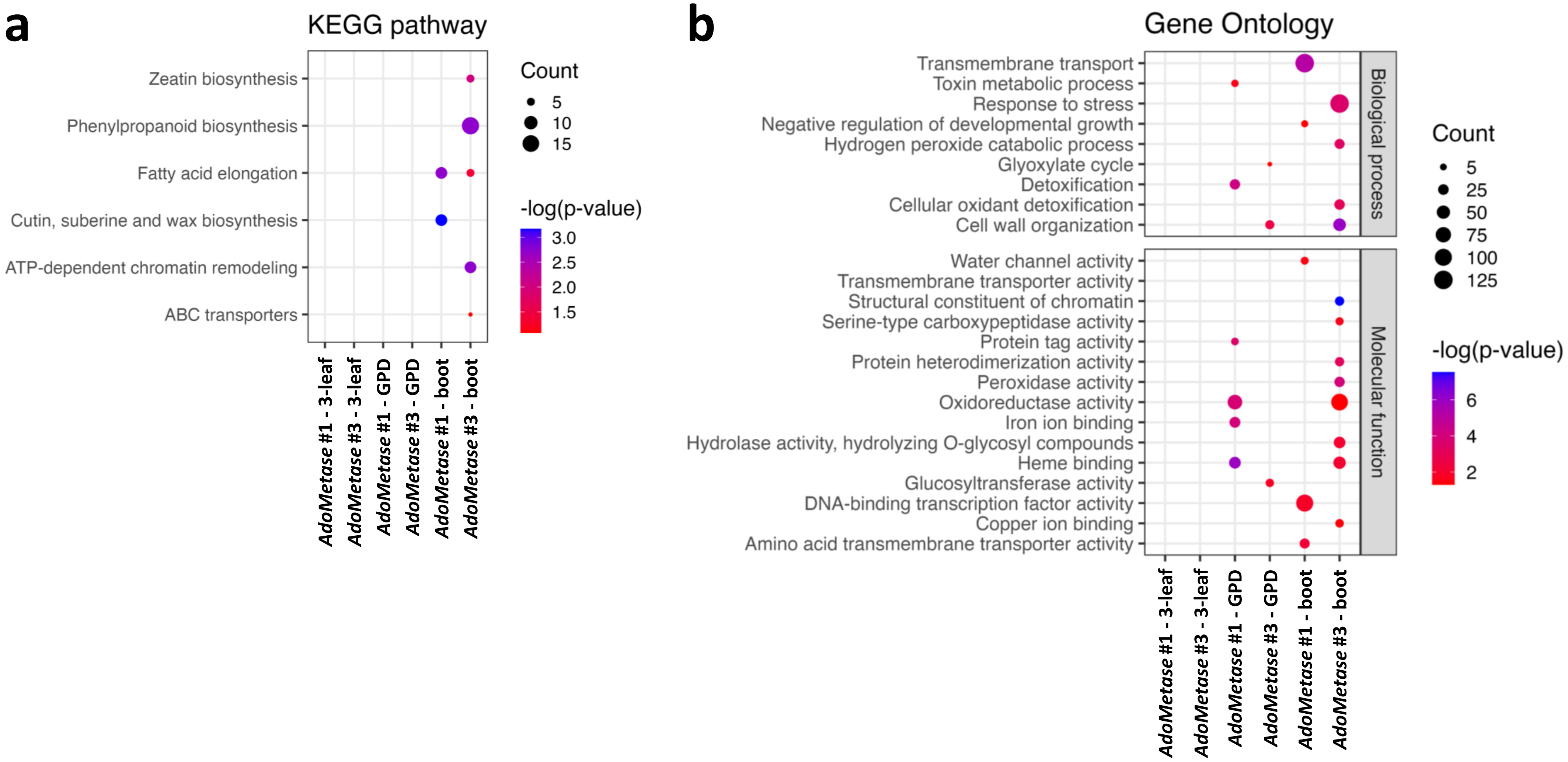

Figure S5: Dot plots of KEGG (**a**) and GO (**b**) enrichment analyses of DEGs identified in *AdoMetase* #1 and #3 at three developmental stages. The size of the dots represents the number of genes associated with each ontology term and pathway.

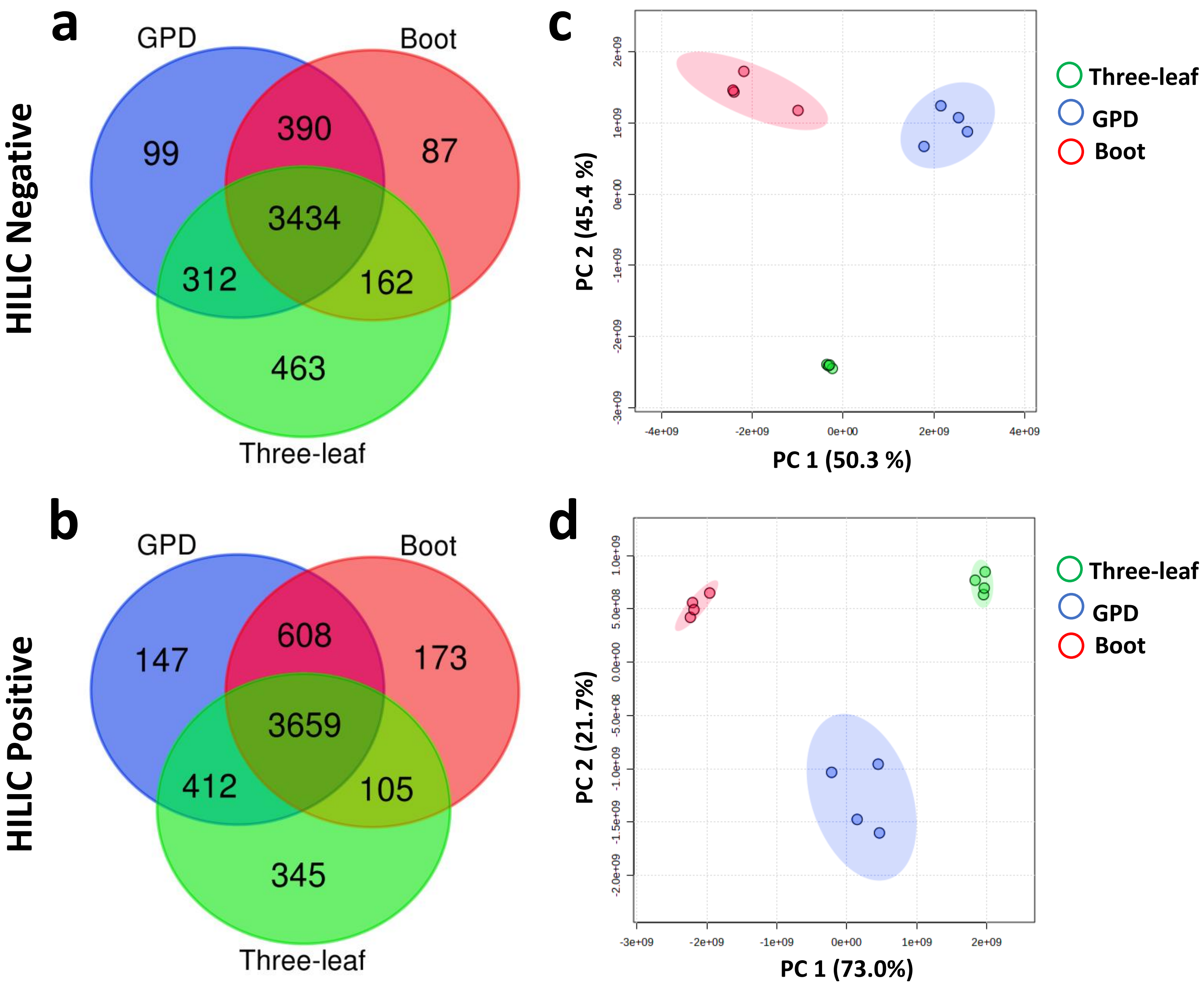

Figure S6: Venn diagrams (**a**-**b**) and PCA plots (**c**-**d**) of the features detected in stems of WT sorghum at three different growth stage using HILIC chromatography.

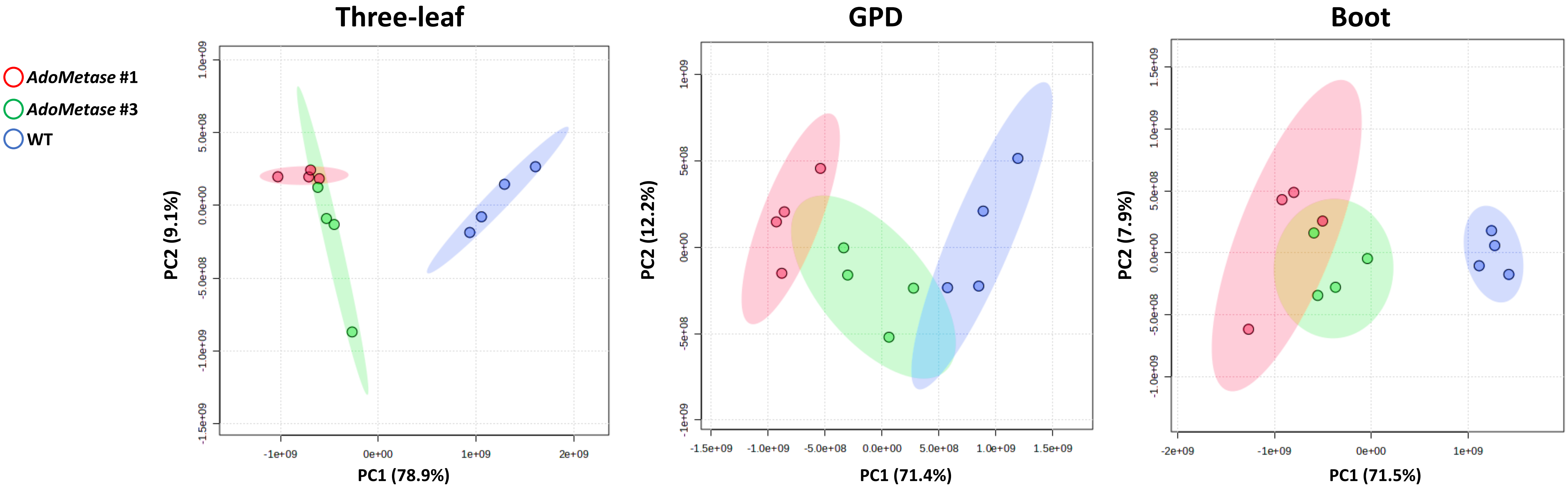

Figure S7: PCA plots of the features detected in stems of WT and *AdoMetase* lines #1 and #3 at three developmental stages using HILIC chromatography (positive ionization mode).

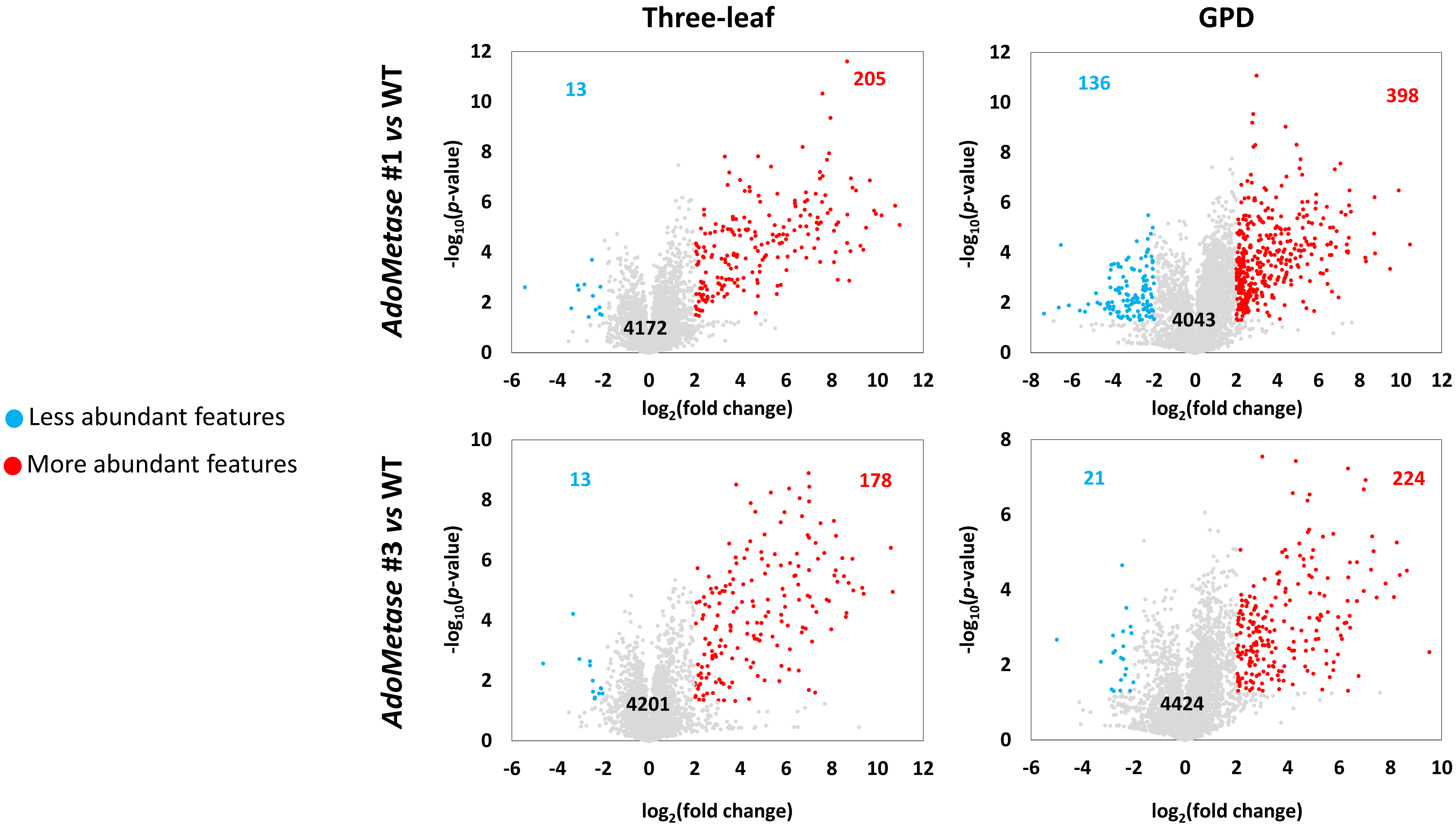

Figure S8: Volcano plots of features detected in stems from WT and transgenic lines at the three-leaf and GPD stages using HILIC chromatography (positive ionization mode). The number of decreased (in blue) and increased (in red) features in *AdoMetase* #1 (upper plots) and *AdoMetase* #3 (lower plots) compared to WT control is indicated on each plot (log_2-fold change +2/-2 and *p*-value < 0.05). Gray dots represent features that are not differentially abundant.

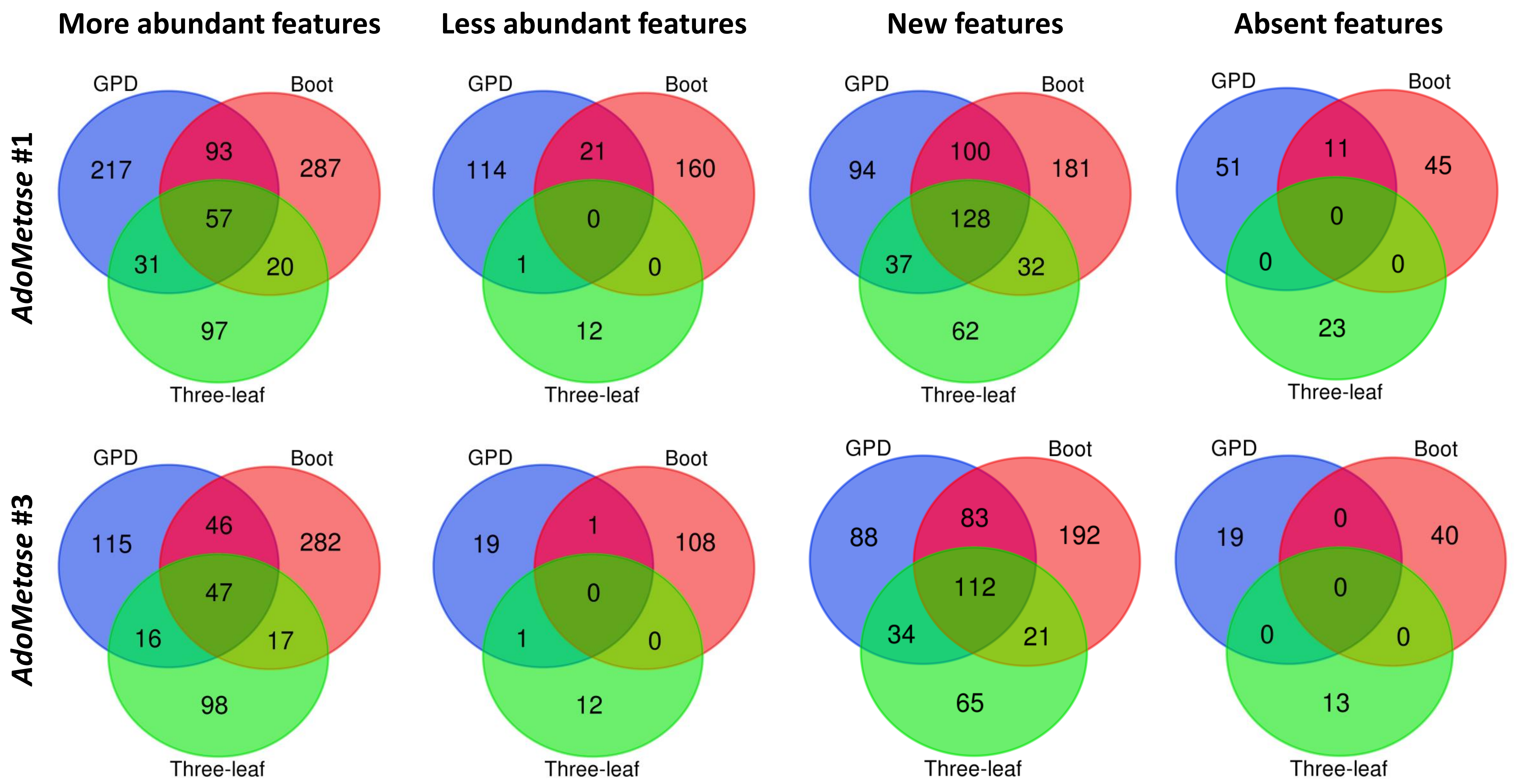

Figure S9: Venn diagrams of the differentially abundant features observed in the *AdoMetase* lines at three different growth stage using HILIC chromatography (positive ionization mode).

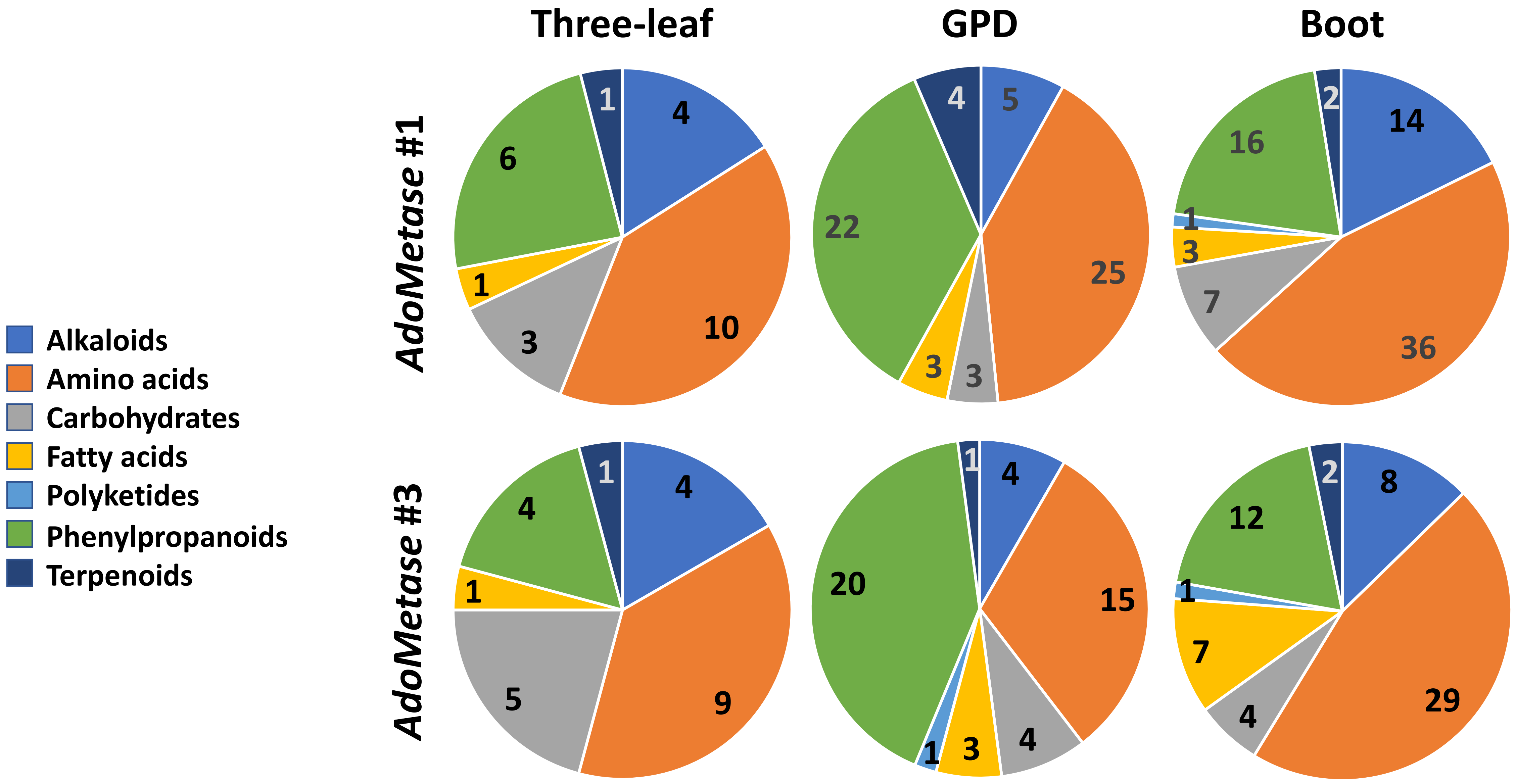

Figure S10: Classification of a subset of differentially abundant metabolites identified in stems of the *AdoMetase* lines at three developmental stages using HILIC chromatography (positive ionization mode). For each class, the number of metabolites is indicated inside the corresponding slice of the pie chart.

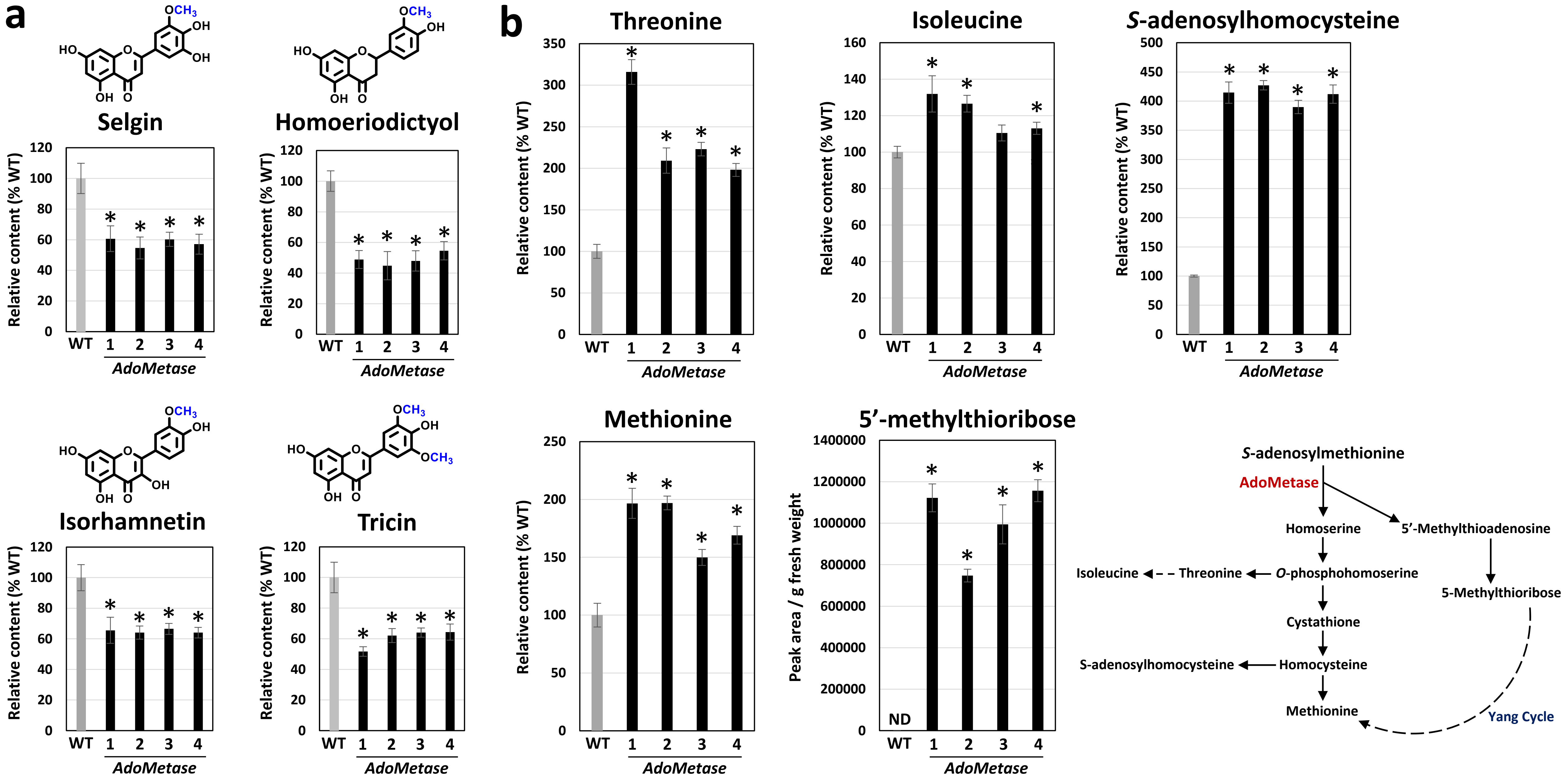

Figure S11: Relative content of targeted metabolites in the *AdoMetase* lines. (**a**) Methylated flavonoids in stem and leaf biomass of mature plants. (**b**) Metabolites derived from the two AdoMetase products homoserine and 5'-methylthioadenosine in 3-week-old seedlings. Asterisks denote significant changes (**P* < 0.05). ND, Not detected. The schematic pathway shows the relationships between metabolites. Dashed arrows denote multiple enzymatic steps. The formation of *S*-adenosylhomocysteine from homocysteine is putative.

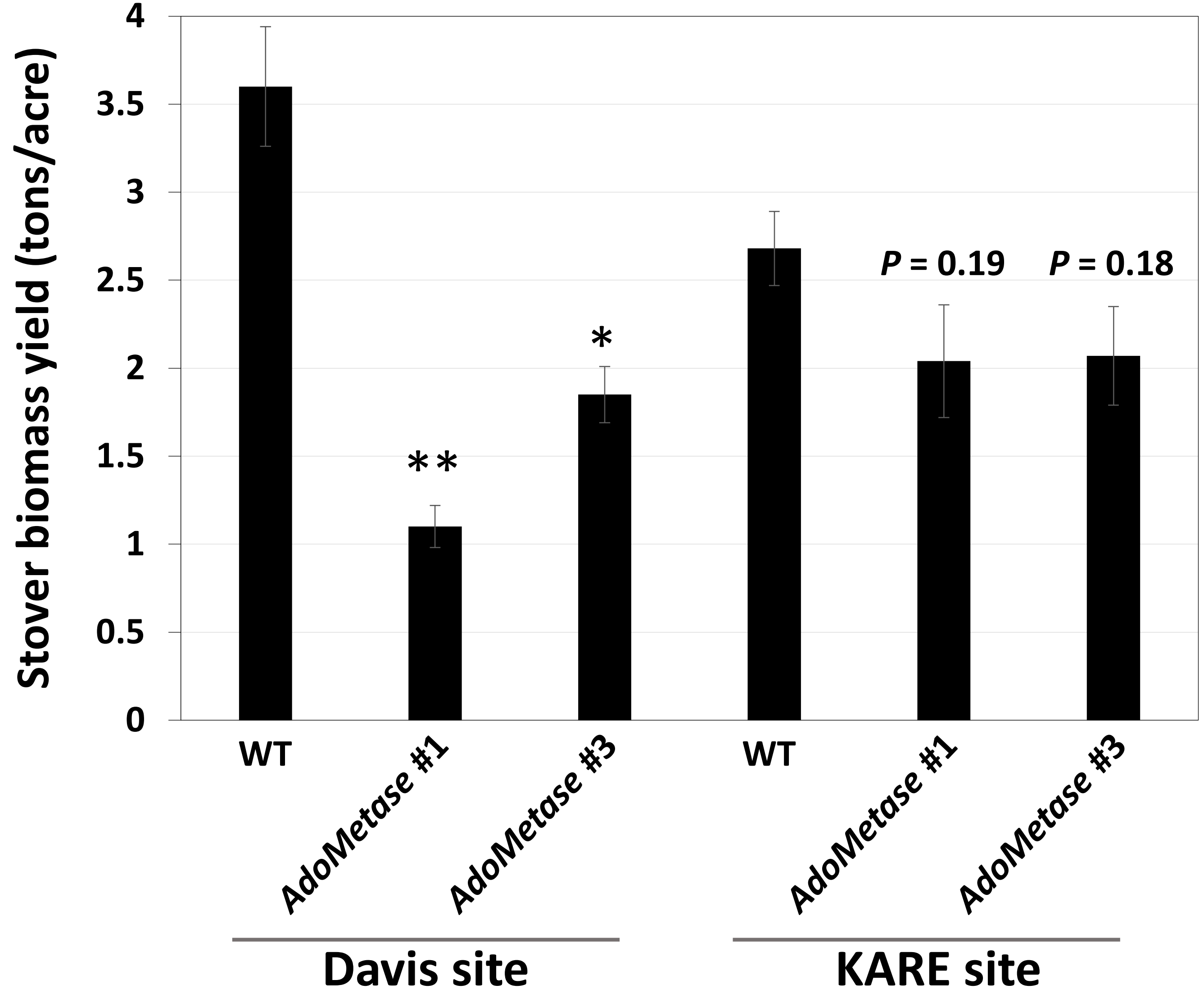

Figure S12: Stover biomass yields from the WT and *AdoMetase* lines grown until the soft dough stage in two different field sites in California. Values are means ±SE of four biological replicates (n = 4 plots). For each site, asterisks indicate significant differences from the WT using the unpaired Student's t-test (*$P < 0.01$, **$P < 0.001$).
